# Collective cancer cell invasion requires RNA accumulation at the invasive front

**DOI:** 10.1073/pnas.2010872117

**Published:** 2020-10-15

**Authors:** George Chrisafis, Tianhong Wang, Konstadinos Moissoglu, Alexander N. Gasparski, Yeap Ng, Roberto Weigert, Stephen J. Lockett, Stavroula Mili

**Affiliations:** ^a^Laboratory of Cellular and Molecular Biology, Center for Cancer Research, National Cancer Institute, NIH, Bethesda, MD 20892;; ^b^Optical Microscopy and Analysis Laboratory, Cancer Research Technology Program, Frederick National Laboratory for Cancer Research, Leidos Biomedical Research Inc. for the National Cancer Institute, NIH, Frederick, MD 21702

**Keywords:** RNA localization, collective invasion, RAB13, NET1, antisense oligo

## Abstract

Localization of RNAs at protrusive regions of cells is important for single-cell migration on two-dimensional surfaces. Protrusion-enriched RNAs encode factors linked to cancer progression, such as the RAB13 GTPase and the NET1 guanine nucleotide exchange factor, and are regulated by the tumor-suppressor protein APC. However, tumor cells in vivo often do not move as single cells but rather utilize collective modes of invasion and dissemination. Here, we developed an inducible system of three-dimensional (3D) collective invasion to study the behavior and importance of protrusion-enriched RNAs. We find that, strikingly, both the *RAB13* and *NET1* RNAs are enriched specifically at the invasive front of leader cells in invasive cell strands. This localization requires microtubules and coincides with sites of high laminin concentration. Indeed, laminin association and integrin engagement are required for RNA accumulation at the invasive front. Importantly, perturbing RNA accumulation reduces collective 3D invasion. Examination of in vivo tumors reveals a similar localization of the *RAB13* and *NET1* RNAs at potential invasive sites, suggesting that this mechanism could provide a targeting opportunity for interfering with collective cancer cell invasion.

## Significance

Specific RNAs are enriched at protrusive regions of migrating cells. This localization is important for cell migration on 2D surfaces. However, in vivo, tumor cells navigate complex 3D environments often in collective groups. Here, we investigated protrusion-enriched RNAs during collective 3D invasion. We show that specific RNAs exhibit a striking accumulation at the front of invasive leader cells. We provide insights into the mechanism underlying RNA accumulation at the invasive front, and we further demonstrate that it is required for efficient 3D invasion of tumor cells. We additionally observe RNA enrichment at invasive sites of in vivo tumors, supporting the physiological relevance of this mechanism and suggesting a targeting opportunity for perturbing cancer cell invasion.

RNA localization, and subsequent localized translation, is emerging as a mechanism required for efficient cell movement. Indeed, localized mRNAs are prominently observed during the process of cell migration. Specifically, RNAs have long been known to accumulate at sites of new integrin engagement (1), at lamellipodia (2, 3), and at sites of spreading or of persistent protrusion formation (4, 5). The number of RNAs enriched at protrusions has significantly expanded through genome-wide screens. Furthermore, RNA accumulation is accompanied by a concentration of various RNA-binding proteins and translation factors at the leading edge and protrusions of migrating cells (6–9). RNA accumulation at protrusions is functionally relevant since preventing protrusion localization of certain RNAs or inhibiting translation at protrusions leads to protrusion destabilization and impedes the efficiency of cell migration (6, 7, 10–12).

Notably, however, to date the roles of protrusion-localized RNAs described above and the underlying mechanisms of localization have been predominantly investigated in single cells migrating on two-dimensional (2D) surfaces, a mode of migration that has been extensively studied. According to the prevalent model, protrusions on 2D surfaces are driven by polymerization of the actin cytoskeleton, which is controlled centrally by Rho GTPase family members. Protrusions are stabilized through adhesions with the extracellular matrix, which also serve as sites of traction as the cell moves forward and are disassembled as the cell contracts at the rear (13, 14). Nevertheless, cells in vivo typically migrate in three-dimensional spaces. In these more complex environments cells come into contact with other cells or with the extracellular matrix, which can present various topographies, mechanical properties, and molecular compositions. In order to navigate such complex surroundings, the cells adopt different modes of migration which can variably depend on Rho-mediated actomyosin regulation and adhesion dynamics (15–17). Therefore, phenomena observed in 2D systems can have variable applicability in in vivo 3D settings.

The ability of cells to migrate in vivo has a central role in cancer metastasis. In this context, emerging evidence suggests that the majority of solid tumors employ a collective mode of migration during invasion and metastasis (18–20). Collectively migrating tumor cells are more aggressive and metastatic than single tumor cells (21, 22), suggesting that this type of migration corresponds to a clinically relevant form of movement. During collective migration, groups of cells move in a concerted manner, remaining connected through cell–cell junctions. In these multicellular structures individual cells adopt different roles by organizing into two functionally distinct groups: leader cells, which are found at the front of invading strands, and follower cells, which remain connected to leaders through mechanical and chemical signaling (19, 23). Here, we set out to understand the behavior and functional contributions of protrusion-localized RNAs during 3D collective invasion.

Among protrusion-localized RNAs we focus on a group of transcripts that are associated with the tumor suppressor protein APC and depend on it for targeting to the cell periphery (5, 6). In particular, we investigate the *RAB13* and *NET1* RNAs. Both RNAs are prominently localized in cell protrusions on 2D surfaces, and deregulation of the corresponding genes has been linked to cancer progression and metastasis (6, 24). Specifically, RAB13 is a member of the Rab family of small GTPases with roles in vesicle-mediated membrane trafficking (25), and its activation at the plasma membrane is required for cell migration and invasion (26). RAB13 expression is amplified in the majority of cancers, and its levels correlate with poor prognosis (25). NET1 acts as a guanine nucleotide exchange factor (GEF) for the RhoA GTPase, a central regulator of cell migration (27). It controls cell motility and has been implicated in the invasion and metastasis of different cancer types (28–30).

Here, using an inducible system of 3D collective invasion, we find that both *RAB13* and *NET1* RNAs are localized in invasive cancer spheroids. This localization is manifested specifically at the front of leader cells in invading cell strands. This pattern of RNA accumulation requires microtubules, laminin association, and integrin engagement. Importantly, perturbing RNA accumulation at the invasive front reduces collective 3D invasion. Furthermore, examination of in vivo tumors reveals a similar accumulation of *RAB13* and *NET1* RNAs at potential invasive sites, suggesting that this mechanism could provide a targeting opportunity for interfering with collective cancer cell invasion.

## Results

### An Inducible Model System of Collective Cancer Cell Invasion.

**Fig. 1.**
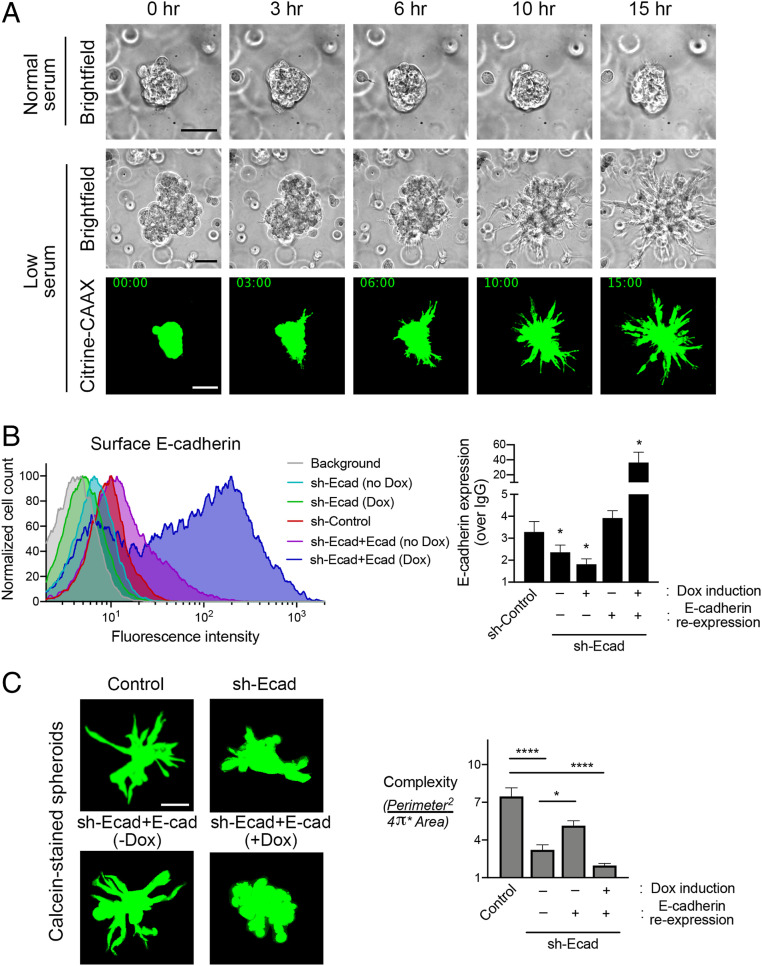
MDA-MB-231 spheroids collectively invade into Matrigel upon serum withdrawal. (*A*) Time-lapse imaging of MDA-MB-231 spheroids embedded in 3D Matrigel. Spheroids were grown and embedded in normal serum (10% fetal bovine serum [FBS]) and switched, or not, to low serum (0.1% FBS) about 4 h after embedding. Time points indicate duration after serum switch. Spheroids of parental cells were visualized with bright-field illumination (*Upper*). Spheroids of cells expressing Citrine–CAAX were visualized with fluorescence microscopy (*Bottom*). (Scale bar, 75 μm.) (*B*) Detection of surface E-cadherin levels through flow cytometry. Example of flow cytometry data (*Left*) and quantification from the indicated cell lines from *n* = 3 independent experiments (*Right*). Background fluorescence (upon staining with IgG) was used to normalize E-cadherin (Ecad) expression. Doxycycline (Dox) was used at 0.1 μg/mL. *P values of 0.048, 0.057, and 0.015, from left to right, by ANOVA with Dunnett’s multiple comparisons test against the sh-Control sample. (*C*) Calcein-stained spheroids of the indicated cell lines embedded in Matrigel, after overnight exposure to low serum (*Left*). Corresponding complexity values (*Right*). *n* = 15 to 24. *P < 0.05, ****P < 0.0001 by ANOVA with Dunnett’s T3 multiple comparisons test. Error bars show SE. (Scale bar, 75 μm.)

To understand the regulation and roles of protrusion-localized RNAs during 3D collective invasion, we developed a system that would allow us to study collective invasion in a controlled manner and simultaneously permit specific RNA imaging. For this, we formed multicellular spheroids of MDA-MB-231 breast cancer cells and embedded them in three-dimensional collagen or Matrigel matrices. In collagen, MDA-MB-231 cells exhibit a highly invasive phenotype. Cells quickly begin invading into the matrix, and individual spheroids become hard to discern within a few hours (*SI Appendix*, Fig. S1*A*). Additionally, the collagen matrix persists during processing for RNA imaging through in situ hybridization and thus interferes with high-quality imaging. In contrast, spheroids embedded in Matrigel, in media with regular serum, remain noninvasive over time (Fig. 1*A*), exhibiting only rotational motions of the whole spheroid structure (Movie S1) (31). Strikingly, however, upon serum withdrawal, Matrigel-embedded spheroids cease rotational movement and become invasive, exhibiting multiple strands of cells penetrating into the surrounding environment (Fig. 1*A* and Movie S2). Serum withdrawal does not lead to any obvious change in the proliferative status of these cells, as assessed by Ki-67 staining (*SI Appendix*, Fig. S1*B*), suggesting that a halt in cell proliferation is not the trigger of invasion in this system. To observe this behavior with higher resolution than that afforded by bright-field imaging, we used MDA-MB-231 cells stably expressing Citrine fluorescent protein carrying a C-terminal CAAX motif. This fluorescent protein is targeted to the membrane, facilitating the delineation of thin cell extensions emanating from the spheroid body. Indeed, we observe again that serum withdrawal induces spheroid invasion (Fig. 1*A* and Movie S3). Additionally, individual cells do not appear to break off the main spheroid body but remain largely in linear strands, reminiscent of collective invasion.

A main characteristic of collective invasion is the maintenance of intercellular contacts through cell–cell junctions, primarily E-cadherin-containing adherens junctions (19, 23). However, in MDA-MB-231 cells we observe expression of mesenchymal markers and low levels of E-cadherin, as previously reported (32). Interestingly E-cadherin expression is increased upon serum withdrawal (*SI Appendix*, Fig. S2*A*), thus suggesting that, despite its low levels, E-cadherin might play a role in the observed induced invasion phenotype. To directly address the role of E-cadherin in this invasion, we knocked down its levels by stable expression of short hairpin RNAs (shRNAs). We then assessed either total E-cadherin expression by Western blot (*SI Appendix*, Fig. S2*B*) or the levels of E-cadherin on the plasma membrane by flow cytometry (Fig. 1*B*). Furthermore, to assess the specificity of any observed effects, we derived lines that reexpress E-cadherin in an inducible manner from a doxycycline-inducible construct. Of note, this exogenous construct resulted in leaky E-cadherin expression even in the absence of doxycycline (*SI Appendix*, Fig. S2*B*, lane 4 and *SI Appendix*, Fig. S2*C*, lane 1). Flow cytometry analysis revealed that leaky reexpression was sufficient to restore plasma membrane–incorporated E-cadherin to almost control levels, while doxycycline addition elevated E-cadherin more than 10-fold with respect to basal levels (Fig. 1*B*). By Western blot, E-cadherin reexpression appeared several fold higher even in the absence of doxycycline (*SI Appendix*, Fig. S2 *B* and *C*), suggesting that a large amount of the reexpressed protein remained in an intracellular compartment, possibly in a nonfunctional form. (We note that while it could be informative to visualize the distribution of E-cadherin in cells within invasive strands, we have been unable to do so because the low level of E-cadherin in these cells is, in our hands, below the detection limit of immunofluorescence assays).

We used this set of knockdown and reexpressing cell lines to assess the role of E-cadherin in spheroid invasion. Spheroids were formed via hanging droplets (see *Materials and Methods*), embedded in Matrigel, and induced to invade by withdrawing serum for ∼18 h. Spheroids were stained with calcein, and a “complexity” metric was calculated (33) to quantitatively assess the degree of invasion (Fig. 1*C*). Interestingly, knockdown of E-cadherin reduced spheroid invasion, supporting the notion that invasion in this system reflects collective invasion. Indeed, leaky E-cadherin reexpression (which restores near-normal E-cadherin levels, in the absence of doxycycline) rescued invasion. Interestingly, higher E-cadherin reexpression, upon doxycycline addition, prevented invasion (Fig. 1*C*). Therefore, overall, MDA-MB-231 cells collectively invade into Matrigel upon serum withdrawal. Despite their mesenchymal character, collective invasion requires E-cadherin and in particular seems to depend on a specific E-cadherin expression range since both knockdown and overexpression abrogate invasion.

### RAB13 and NET1 RNAs Localize at the Front of Invasive Leader Cells.

**Fig. 2.**
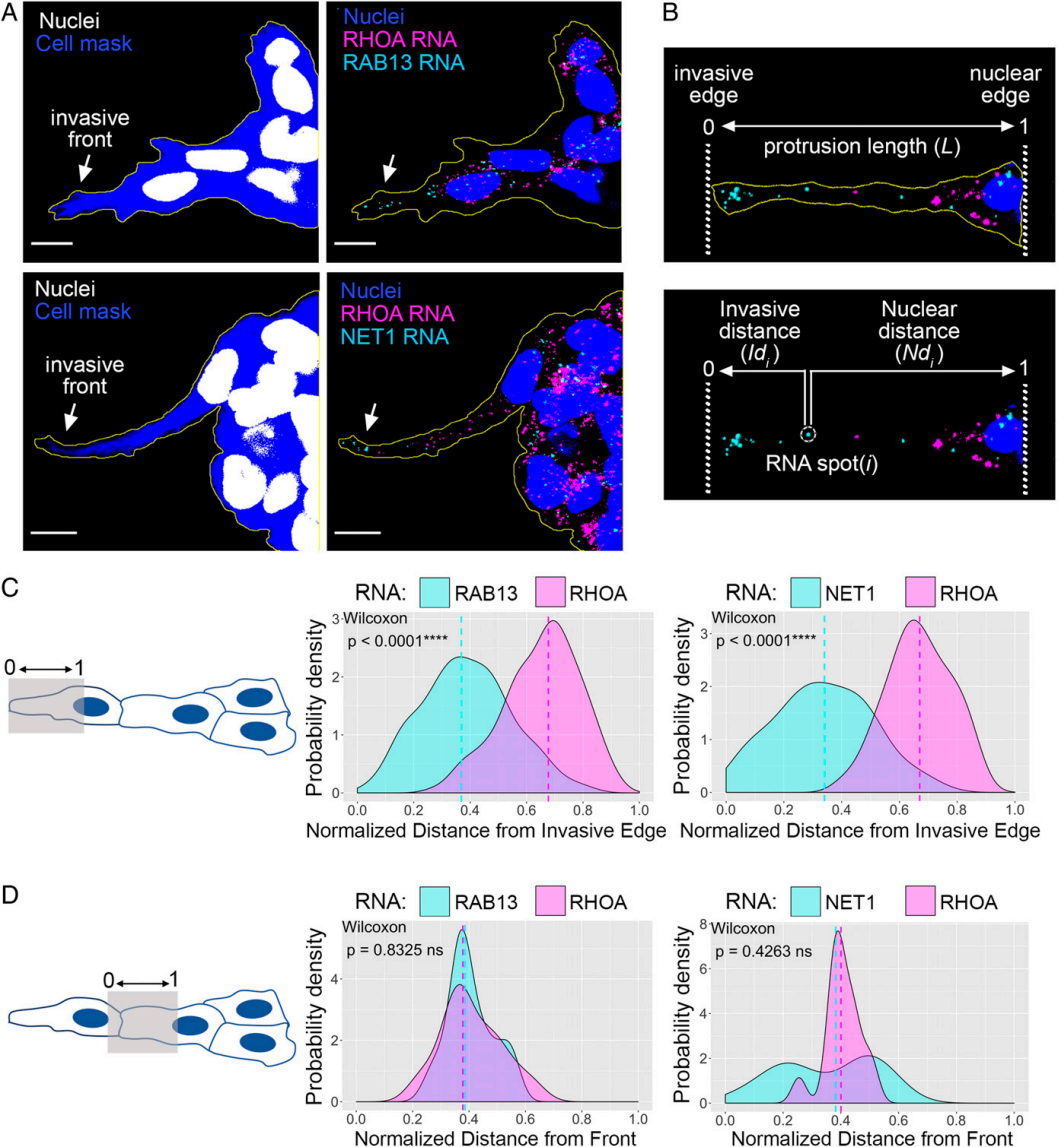
*RAB13* and *NET1* RNAs localize at the front of invasive leader cells. (*A*) In situ hybridization of invasive MDA-MB-231 spheroids in Matrigel. The indicated RNAs were detected, and cell mask staining was used to delineate the spheroid outline. Arrows point to *RAB13* and *NET1* RNA accumulation at the invasive front. (Scale bar, 10 μm.) (*B*) Representative image of the front cytoplasm of a leader cell used for quantitative analysis. Invasive and nuclear edges are manually defined, and for each RNA spot *i*, its distance from the invasive edge (*Id*_*i*_) and nuclear edge (*Nd*_*i*_) is obtained. The total protrusion length (L) is derived by summing *Id*_*i*_ and *Nd*_*i*_. For each RNA species (e.g., *RAB13* or *RHOA*) the average RNA distance is then computed and normalized between values of 0 (invasive edge) and 1 (nuclear edge) (see *Materials and Methods* for details). (*C*) Quantification of RNA distributions within the front cytoplasm of leader cells (shaded area in *Left* schematic). Average distance values, calculated as described in *B*, were used to derive the probability density function of the indicated RNAs. The *RHOA* RNA is used as an internal control in each case. *n* = 125 (*RAB13-RHOA*), *n* = 42 (*NET1-RHOA*); from a minimum of three independent experiments. (*D*) Analysis as in *C* focusing on the cytoplasm between the leader and follower cell nuclei (shaded area in *Left* schematic). Note that because in some images the rear cytoplasm of the leader cell is included in the analysis, this can skew the values toward the front, as noticeably seen in the *Right* graph. Comparisons are performed against the internal control *RHOA* RNA, which is detected in the same cells and is subject to the same segmentation limitations. *n* = 25 (*RAB13-RHOA*), *n* = 14 (*NET1-RHOA*); from a minimum of three independent experiments. *P* values determined by the Wilcoxon signed-rank test; ns, not significant.

To assess RNA distributions in collectively invading spheroids, we used single-molecule in situ hybridization. We focused on the *RAB13* and *NET1* RNAs, two protrusion-localized RNAs which encode proteins with roles in cell migration (see Introduction). As an internal control, we concomitantly detected the *RHOA* RNA, which shows a diffuse cytoplasmic distribution. During fixation and processing, the majority of Matrigel dissolves, allowing probe penetration and high-resolution imaging. Indeed, RNAs can be detected throughout the spheroid body (*SI Appendix*, Fig. S3 *A* and *B*), and as expected, most of the signal is detected in the cell cytoplasm (*SI Appendix*, Fig. S3 *B*, *Right*). When we focused on invasive cell strands, we strikingly observed that both the *RAB13* and *NET1* RNAs accumulate prominently at the front of the invasive leader cell, while the *RHOA* RNA exhibits a more perinuclear or diffuse distribution with few RNAs reaching the invasive edge (Fig. 2*A*).

To quantitatively assess RNA distributions in leader cells, we first focused on the front cytoplasm, defined as the cytoplasm between the leader cell nucleus and invasive tip. For each image, a spot detection algorithm was used to identify individual RNA spots and calculate their distance from the corresponding invasive and nuclear edges (Fig. 2*B*). To evaluate RNA distributions across multiple cells, all RNA distances were first normalized between 0 and 1, where 0 corresponds to the invasive edge and 1 corresponds to the nuclear edge. Then, for every cell, an average, normalized RNA distance was computed, and average values from multiple images were used to derive and plot the probability density function of specific RNAs within the front cytoplasm of leader cells (Fig. 2*C*; see *Materials and Methods* for details). Both the *RAB13* and *NET1* RNAs showed a clearly biased distribution toward the invasive front, and in both cases, this was significantly different from the *RHOA* distribution, which was centered closer to the nucleus (Fig. 2*C*).

To evaluate whether this preferential enrichment toward the invasive front is specifically occurring in leader cells, we performed a similar analysis focusing on the cell immediately following each leader (Fig. 2*D*). Since it is not possible to precisely identify the cell boundaries, this analysis includes portions of the rear cytoplasm of the leader cell. However, we reasoned that the comparison to an internal control RNA (i.e., *RHOA*) would allow us to discern differences in distributions. We observe that in these “follower” cells, both the *RAB13* and *NET1* RNAs exhibit a distribution that cannot be distinguished from that of *RHOA,* with almost identical mean values (Fig. 2*D*). Therefore, taking all the above evidence together, we conclude that the *RAB13* and *NET1* RNAs become enriched at the invasive front during collective 3D invasion, and this behavior appears to selectively occur in leader cells.

### RNA Localization at the Invasive Front Requires Microtubules.

**Fig. 3.**
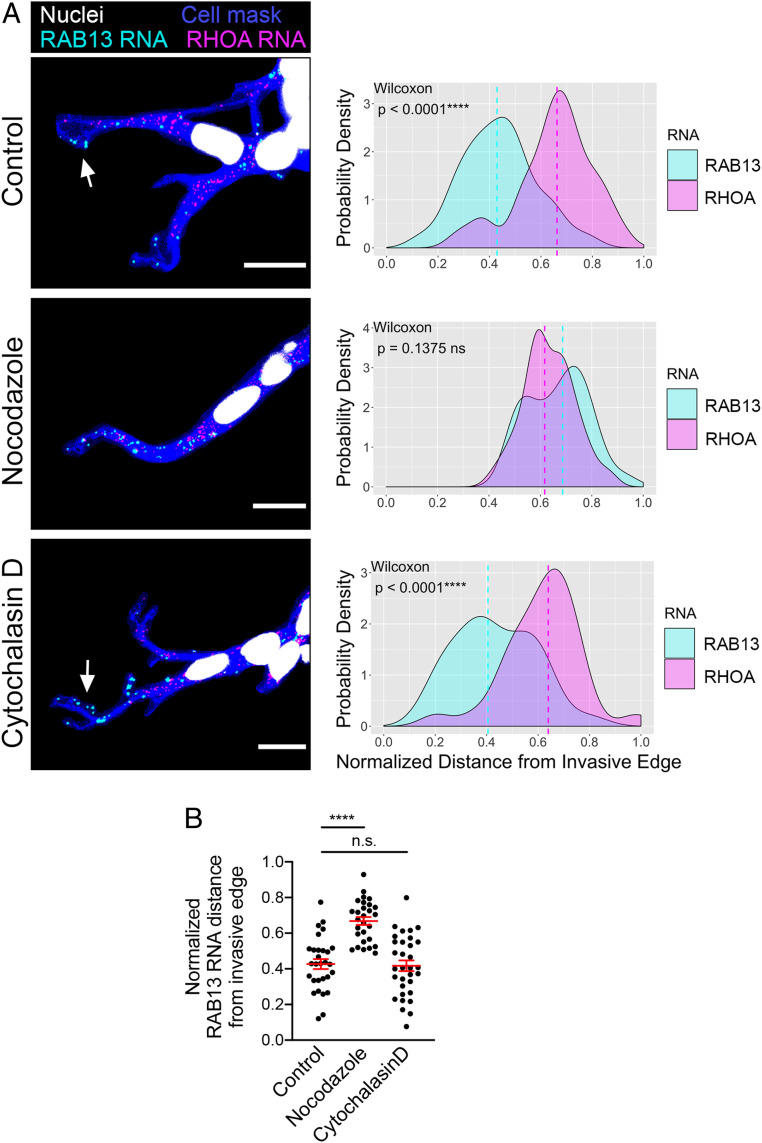
RNA localization at the invasive front requires microtubules. (*A*) Representative in situ hybridization images detecting *RAB13* and *RHOA* RNAs within leader cells of invasive spheroids treated for 45 min with the indicated compounds. Associated probability density plots are derived from *n* = 28 to 29 images, two independent experiments. *P* values determined by the Wilcoxon signed-rank test. (Scale bar, 15 μm.) (*B*) Comparison of normalized *RAB13* RNA distances. ****P < 0.0001; ns, nonsignificant by ANOVA with Dunnett’s multiple comparisons test. Error bars show SE.

Cytoskeletal elements are required for localization of RNAs at the protrusions of cells migrating on two-dimensional surfaces (6, 9, 34). To understand if a similar mechanism underlies the localization of RNAs at the invasive front during 3D invasion, we imaged RNAs in invading spheroids after brief treatment with nocodazole or cytochalasin D. Depolymerization of microtubules with nocodazole (*SI Appendix*, Fig. S4) disrupted *RAB13* RNA accumulation at the invasive front (Fig. 3*A*). By contrast, interference with the actin cytoskeleton by cytochalasin D (*SI Appendix*, Fig. S4) did not appreciably alter *RAB13* RNA localization. These effects were observed when comparing *RAB13* to the internal *RHOA* control (Fig. 3*A*) as well as when comparing the normalized *RAB13* RNA distances between conditions (Fig. 3*B*). Thus, the microtubule cytoskeleton is required for persistent RNA accumulation at the invasive front.

While we have not directly tested the mechanisms underlying localization of the *NET1* RNA in this 3D system, we consider it likely that it is regulated similarly to *RAB13,* given that in 2D systems the two RNAs are regulated similarly with regard to their dependence on microtubules, accumulation in the same type of protrusions, association with protein factors, and coexistence in the same multimeric RNA granules (6, 24).

### Laminin Accumulation and Integrin Engagement at the Invasive Front Promote RNA Localization.

**Fig. 4.**
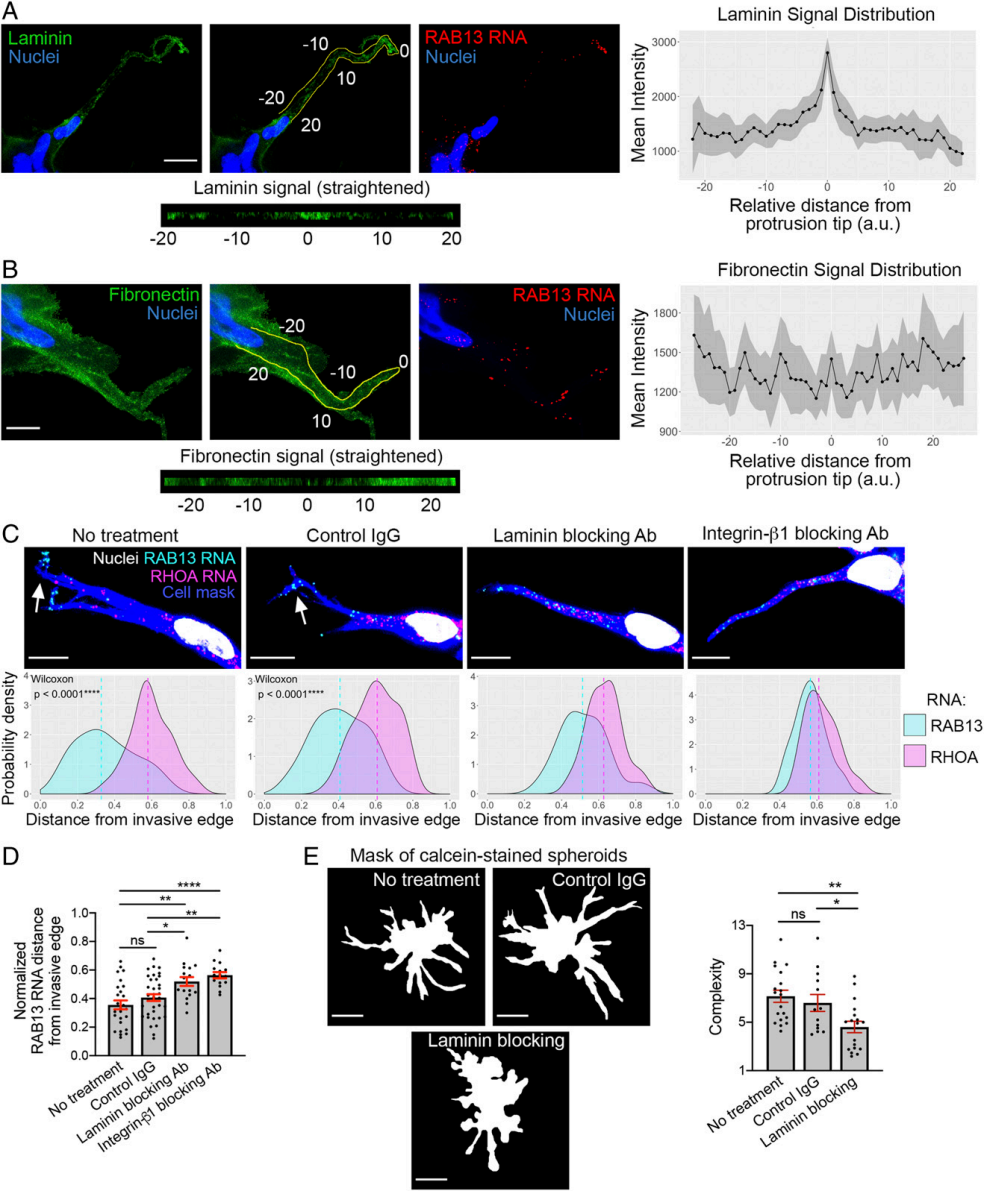
Laminin accumulation and engagement at the invasive front promote RNA localization. (*A*) Laminin distribution around leader cells of invasive spheroids. (*Left* image panels) Laminin immunostaining and *RAB13* RNA detection focusing on a leader cell. The middle panel on the *Left* depicts the perimeter along the front cytoplasm used to quantify laminin intensity; 0 indicates the tip of the leader cell protrusion. The corresponding signal is presented straightened in the *Bottom*. (Scale bar, 15 μm.) (*Right* graph) Mean laminin intensity along the perimeter of leader cells. Distances are relative to the tip, which is set at 0. Shaded area indicates 95% CI. *n* = 23. (*B*) Fibronectin distribution around leader cells. Analysis as in *A*. *n* = 28. (Scale bar, 10 μm.) (*C*) Representative in situ hybridization images detecting *RAB13* and *RHOA* RNAs within leader cells of invasive spheroids treated with the indicated blocking antibodies for 3 h. (Scale bar, 10 μm.) Associated probability density plots are derived from *n* = 16 to 36 images. *P* values determined by the Wilcoxon signed-rank test. (*D*) Comparison of normalized *RAB13* RNA distances. (*E*) Calcein-stained spheroids treated overnight with the indicated antibodies and corresponding complexity values. *n* = 13 to 20. *P < 0.05, **P < 0.01, ****P < 0.0001; ns, nonsignificant by ANOVA with Tukey’s multiple comparisons test. Error bars show SE. (Scale bar, 70 μm.)

Localization of RNAs is additionally influenced by engagement with and the properties of the extracellular matrix. For example, the stiffness of the environment and engagement of integrins promote peripheral RNA accumulation (1, 6). We therefore investigated whether the extracellular matrix at the invasive front could participate in the observed local RNA accumulation. Since laminin is a major constituent of Matrigel, we detected laminin through immunofluorescence. Interestingly, we observed an accumulation of laminin at the tip of the invasive front (Fig. 4*A*). Indeed, quantification of the laminin signal intensity along the perimeter of the front cytoplasm of leader cells (Fig. 4 *A*, *Left,* *Center*) revealed that significantly more laminin was present at the invasive tip than in any other rearward position (Fig. 4 *A*, *Right*). By contrast, fibronectin was uniformly found along the leader cell, with only apparently random variations in intensity (Fig. 4*B*).

This increased laminin accumulation could be indicative of denser or stiffer laminin organization. Pertinent, in this context, is the fact that the localization of *RAB13* and other protrusion-enriched RNAs is regulated by the stiffness of the extracellular environment (6). Combined with the observation that laminin accumulation at the invasive tip coincides with the site of intracellular *RAB13* RNA localization (Fig. 4 *A*, *Right*), these findings raised the possibility that laminin sensing and engagement by the leader cell could have a causal role in driving RNA localization at the invasive front. To directly test this hypothesis, we induced spheroid invasion in the presence of a laminin-blocking antibody and assessed RNA localization in leader cells (Fig. 4 *C* and *D*). Additionally, we blocked integrin-β1 using a respective blocking antibody since integrin-β1 is part of the integrin receptor for the main laminin-1 isoform in Matrigel (35). However, prolonged (24 h) treatment in the presence of the integrin-blocking antibody (Ab) severely hindered invasion (*SI Appendix*, Fig. S5*A*), preventing the identification of leader cells. To first find conditions that would allow us to assess the effect of integrin blocking on RNA localization, we induced spheroid invasion, added the blocking antibody, and imaged spheroids at various time points to identify a duration of exposure that would enable the identification and imaging of leader cells (*SI Appendix*, Fig. S5*B* and Movie S4 [treatment with integrin-blocking antibody] and Movie S5 [treatment with control antibody]). Based on these data, a 3 h antibody treatment was selected. At this time, while some protrusions have started stalling, there is no overt retraction. Laminin blocking does not impede invasion as severely as integrin blocking but, nevertheless, significantly reduces it, after overnight treatment, as assessed by spheroid complexity measurements (Fig. 4*E*). Such a partial effect is rather anticipated given the large amount of laminin present in Matrigel. Under these Ab-blocking conditions we then imaged RNAs within leader cells. Importantly, these experiments revealed that blocking laminin and integrin engagement significantly shifted the *RAB13* RNA away from the invasive front (Fig. 4*D*) and toward a more perinuclear distribution (Fig. 4*C*). We cannot unequivocally exclude that extracellular matrix (ECM) engagement is primarily required for protrusion formation, which in turn leads to RNA accumulation. Nevertheless, we think the fact that we can detect loss of RNA localization even before the beginning of overt retraction rather suggests that persistent RNA accumulation at the front of invasive leader cells directly requires ECM engagement.

### RAB13 RNA Localization at the Invasive Front Is Required for Collective Invasion.

**Fig. 5.**
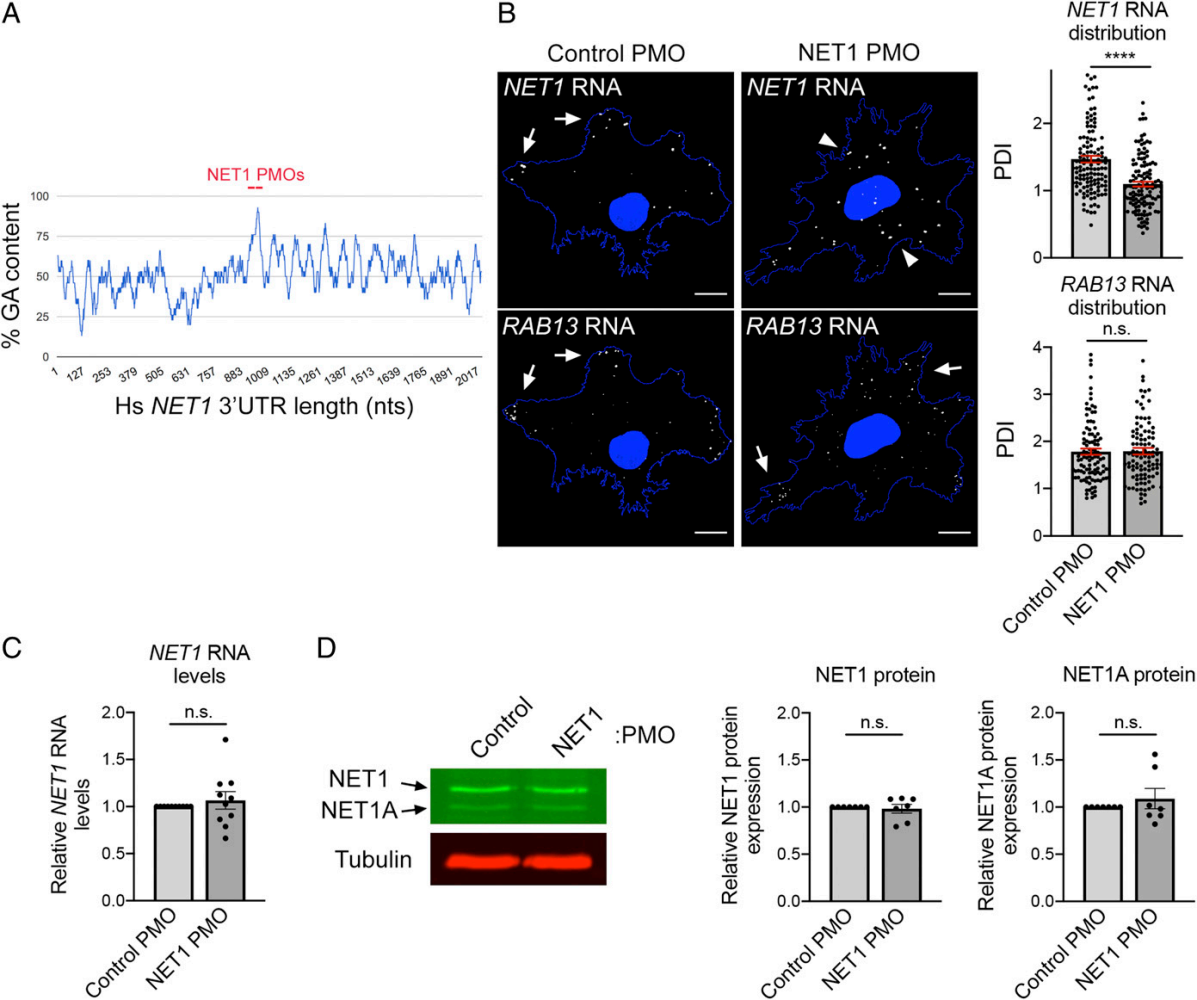
Antisense oligonucleotides against a GA-rich region in the NET1 3′UTR specifically interfere with the localization of *NET1* RNA. (*A*) Schematic showing the percent GA content along the human *NET1* 3′UTR (using a 30 nucleotide [nt] window size) and the positions targeted by antisense PMOs. (*B*) FISH images of *NET1* and *RAB13* RNAs in cells treated with the indicated PMOs on a 2D surface. Arrows indicate peripheral RNA; arrowheads indicate perinuclear RNA. Corresponding graphs depict Peripheral Distribution Index (PDI) values measuring peripheral RNA distribution. A PDI value of 1 indicates a diffuse distribution. Note that the *NET1* RNA becomes more perinuclear in cells treated with NET1 PMOs, while the *RAB13* RNA remains peripherally localized. (Scale bar, 15 μm.) (*C*) *NET1* RNA levels measured by droplet digital PCR (ddPCR) analysis. (*D*) Representative Western blot analysis to assess NET1 protein levels and corresponding quantifications. Note that two NET1 isoforms exist (NET1 and NET1A). ****P < 0.01; ns, nonsignificant by the Mann–Whitney *U* test (*B*) or Wilcoxon signed-rank test (*C* and *D*).

The above experiments additionally revealed a correlation between RNA enrichment at the front of leader cells and overall spheroid invasiveness (Fig. 4 *C*–*E* and *SI Appendix*, Fig. S5), hinting toward a potential functional link between them. It is possible that RNA localization either functionally contributes to 3D invasion or that it is a secondary byproduct of it. To distinguish between these two possibilities and determine if disrupting RNA enrichment is sufficient to perturb invasion, we employed a method that allows us to prevent the localization of individual RNAs to protrusions. In particular, we recently identified a GA-rich region in the 3′UTR of the *RAB13* RNA that is important for localization and designed antisense phosphorodiamidate morpholino oligonucleotides (PMOs) that are targeted against it (11). Delivery of these antisense RAB13 PMOs specifically prevents the localization of the *RAB13* RNA at protrusions without affecting *RAB13* RNA stability or translation (RAB13 PMO characterization is detailed in ref. 11). We have additionally determined here that targeting a GA-rich region within the *NET1* 3′UTR similarly disrupts the accumulation of *NET1* RNA in peripheral protrusions on 2D surfaces (Fig. 5 *A* and *B*). The nontargeted *RAB13* RNA remains peripherally enriched under these conditions. Importantly, NET1 PMOs do not alter the overall amount of *NET1* RNA or the amount of NET1 protein produced (Fig. 5 *C* and *D*). Therefore, this methodology enables the assessment of the functional effects mediated exclusively by the *RAB13* or *NET1* RNA localization.

**Fig. 6.**
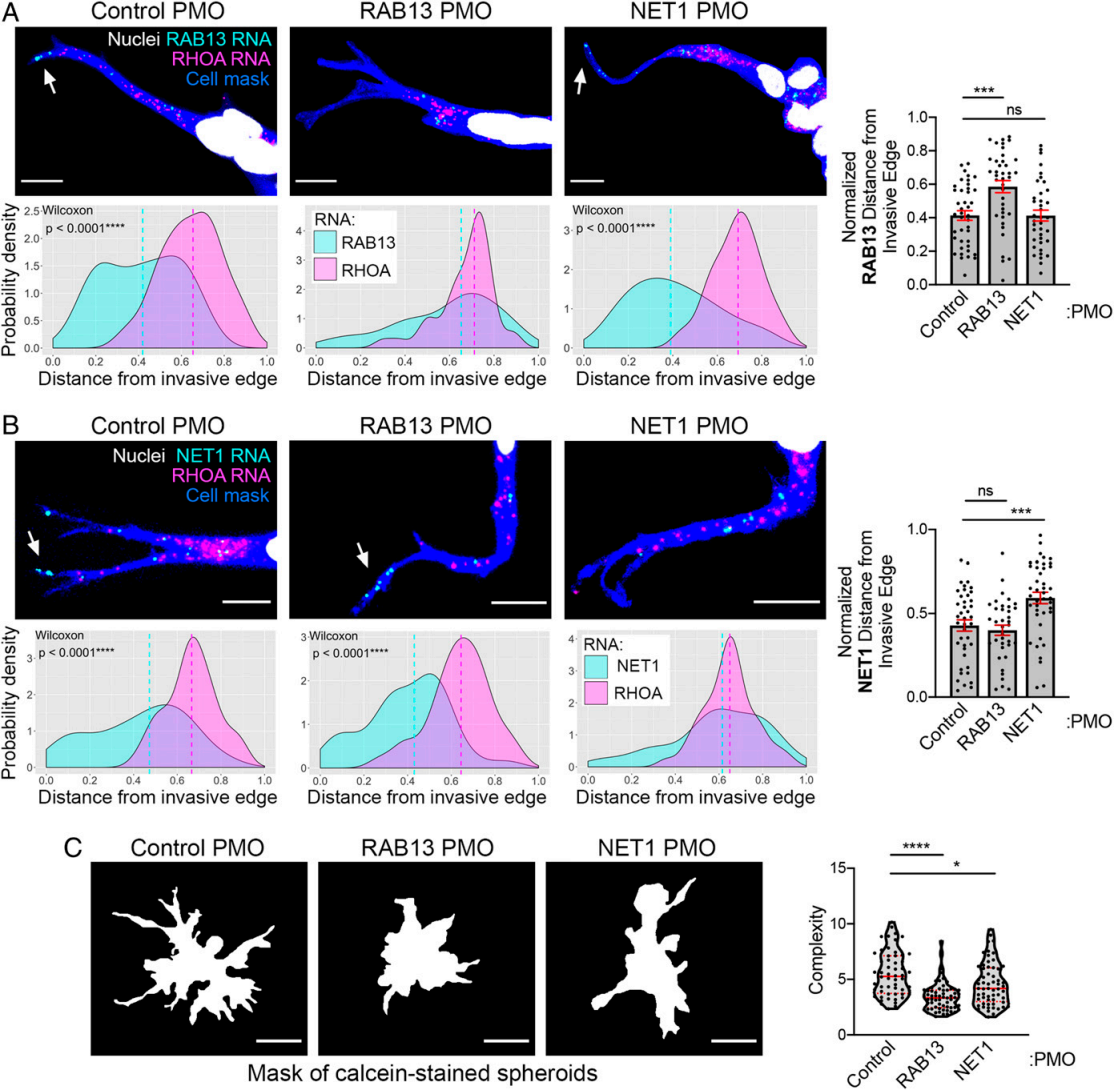
*RAB13* and *NET1* RNA localization at the invasive front is required for collective invasion. (*A* and *B*) Representative in situ hybridization images detecting *RAB13* and *RHOA* RNAs (*A*) or *NET1* and *RHOA* RNAs (*B*) within leader cells of invasive spheroids treated with the indicated PMOs. (Scale bar, 10 μm.) Associated probability density plots are derived from *n* = 37 to 42 images, three independent experiments. *P* values determined by the Wilcoxon signed-rank test. If not indicated, then differences are not significant. *Right* graphs depict comparison of normalized *RAB13* RNA (*A*) or *NET1* RNA (*B*) distances between PMO-treated spheroids. (*C*) Calcein-stained spheroids treated with the indicated PMOs and corresponding complexity values. (Scale bar, 70 μm.) *n* = 56 to 63, three independent experiments. *P <0.05, ***P < 0.001, ****P < 0.0001; ns, nonsignificant by the Kruskal–Wallis with Dunn’s multiple comparisons test (*A* and *C*) or ANOVA with Dunnett’s multiple comparisons test (*B*). Error bars show SE. Red lines in violin plots indicate median and quartile values.

We employed this methodology to assess whether *RAB13* or *NET1* RNA accumulation at the invasive front is required for collective invasion. We first determined whether PMOs against the GA-rich region of the *RAB13* or *NET1* 3′UTRs were able to interfere with localization of the RNA in this 3D setting. PMOs can be efficiently delivered in >90% of MDA-MB-231 cells, and they persist in cells for at least 3 d (11), allowing sufficient time for spheroid formation and induction of invasion. Indeed, RNA imaging in leader cells revealed that RAB13 or NET1 PMOs altered the distribution of their respective RNA targets, making them more perinuclear and increasing the average RNA distance from the invasive edge (Fig. 6 *A* and *B*). Notably, in each case, the distribution of the nontargeted RNAs in leader cells was not affected (Fig. 6 *A* and *B*), attesting to the specificity of the approach.

Importantly, complexity measurements showed that spheroids treated with RAB13 or NET1 PMOs exhibit significantly reduced complexity*,* indicative of reduced invasiveness (Fig. 6*C*). However, given that there is a 3 d lag between PMO delivery and invasion assessment, we wanted to exclude the possibility that PMOs are affecting cell growth and potentially resulting in the formation of smaller spheroids composed of fewer cells, which might be expected to have relatively lower complexity values. Nevertheless, we do not detect any effect of PMO delivery on cell growth rates over multiple days (*SI Appendix*, Fig. S6*A*). Furthermore, we compared complexity values among spheroids of similar areas and detected a consistent decrease in complexity for PMO-treated spheroids, regardless of spheroid size (*SI Appendix*, Fig. S6*B*). We conclude that localization of the *RAB13* or *NET1* RNA at the front of invasive leader cells contributes directly to and is necessary for efficient 3D collective invasion.

### RAB13 and NET1 RNAs Accumulate at Potential Invasive Sites in In Vivo Tumors.

**Fig. 7.**
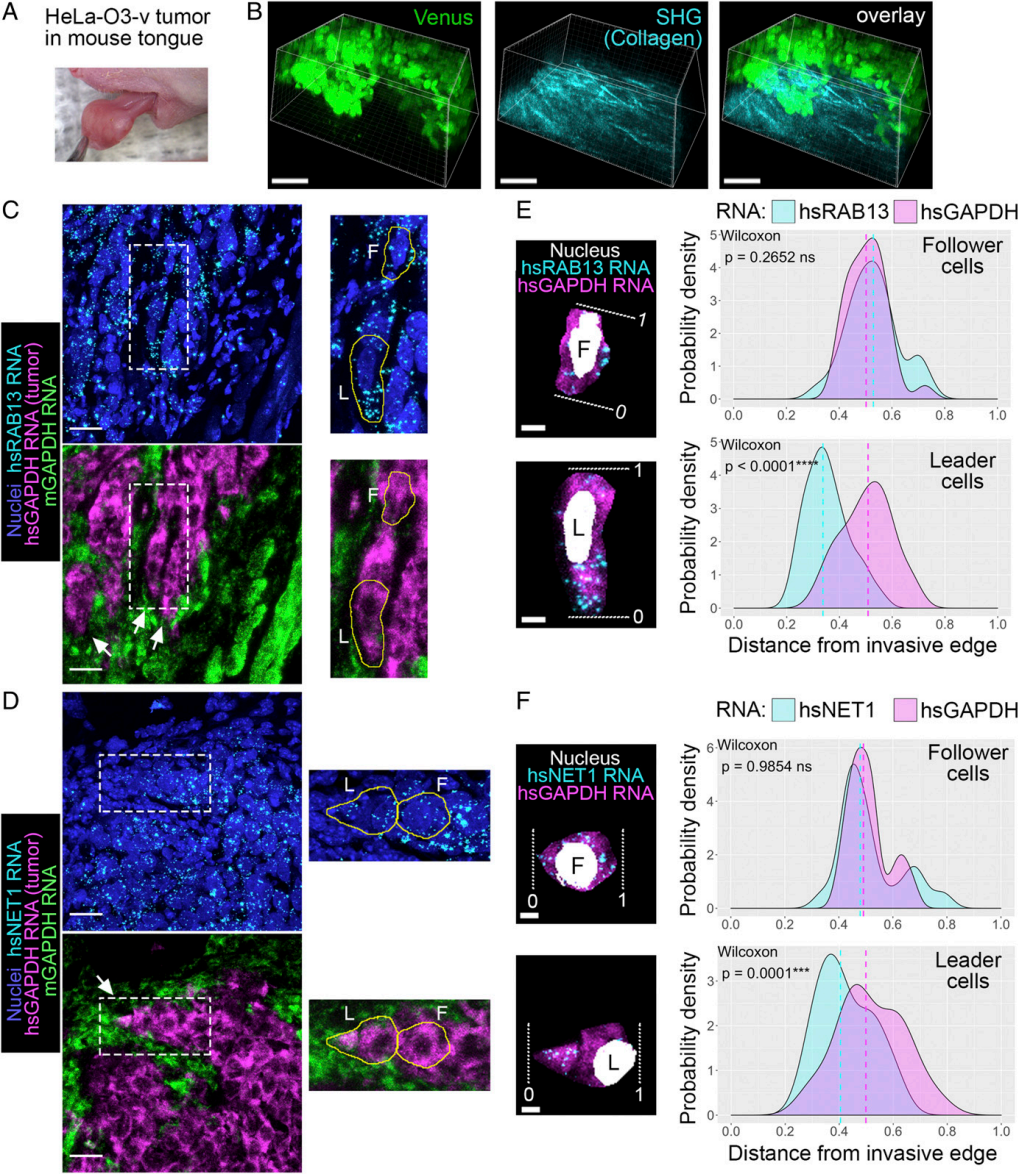
*RAB13* and *NET1* RNAs accumulate at potential invasive sites in in vivo tumors. (*A*) Xenograft tumor mass of HeLa-O3-v cells 2 wk after injection in a mouse tongue. (*B*) Intravital fluorescence imaging of tumor mass. Tumor cells are visualized through Venus fluorescence. Collagen fibers are visualized through second-harmonic generation (SHG). (Scale bars, 100 μm.) (*C* and *D*) Tongue tissue sections (20 μm thick) were processed to detect the indicated RNAs. Species-specific *GAPDH* RNA probes were used to delineate the tumor cells (hsGAPDH) from the normal mouse tissue (mGAPDH). White arrows point to potential invasive strands of tumor cells. *Insets* are magnified to the *Right* with leader (L) and follower (F) cells outlined. (Scale bars, 20 μm.) (*E* and *F*) Isolated leader and follower cells indicated in *C* and *D*. White dashed lines indicate the invasive front (0; for the follower cell, 0 indicates the edge facing toward the invasive front) or the rear of the cell (1). Probability density plots of indicated RNA distributions are derived from *n* = 29 cells (RAB13) or *n* = 20 cells (NET1) from two animals. *P* values determined by Wilcoxon signed–rank test; ns, not significant. (Scale bars, 5 μm.)

To address the generality and potential physiological relevance of this mechanism, we tested whether this RNA accumulation phenomenon can be observed in invasive tumors in vivo. For this we used an established tumor xenograft model in which human cancer cells are injected into the tongue of immunocompromised mice (36). We used HeLa-O3-v cells, a highly invasive variant of HeLa adenocarcinoma cells which express Venus fluorescent protein. Similar to MDA-MB-231 cells, HeLa-O3-v cells exhibit prominent localization of both *RAB13* and *NET1* RNAs at protrusive cytoplasmic regions (*SI Appendix*, Fig. S7). Of note, when embedded in vitro in 3D collagen or Matrigel matrices, spheroids of HeLa-O3-v cells do not exhibit an invasive phenotype (*SI Appendix*, Fig. S8), suggesting that the in vitro conditions that trigger invasion vary depending on the particular cell type. Nevertheless, injection of these cells into mouse tongue results in the formation of a visible tumor mass within 1 to 2 wk (Fig. 7 *A* and *B*). These tumor masses are highly invasive since tumor cells can be detected in cervical lymph nodes in the majority (>80%) of cases within 3 wk of injection (36).

We investigated the distribution of the *RAB13* and *NET1* RNAs in these invasive tumor masses by performing in situ hybridization of tumor tissue sections. We used species-specific probes against the human or mouse housekeeping *GAPDH* RNA to discriminate human tumor cells from normal mouse cells within each tissue section. We further detected the human *RAB13* or *NET1* RNAs within the tumor mass (Fig. 7 *C* and *D*). The staining was highly specific, with no background signal observed when negative control RNA probes, which target a bacterial gene (dapB), were used (*SI Appendix*, Fig. S9). *GAPDH* staining allowed the identification of tumor boundaries, which was also corroborated by differences in nuclei size, with human cells having larger and rounder nuclei than the surrounding normal mouse tissue (Fig. 7*C*). At tumor boundaries we could often identify strands of tumor cells penetrating into the surrounding normal tissue (Fig. 7 *C* and *D*; white arrows), which likely correspond to collectively invading strands. Within such strands we focused on the front leader cell or on a follower cell within the same strand (marked L and F respectively in the *Insets* of Fig. 7 *C*–*F*). As described above, we analyzed the probability density distribution of the *RAB13* or *NET1* RNAs along the cell axis from the invasive front (or the edge facing toward the invasive front) to the back of the cell (Fig. 7 *E* and *F*). Remarkably, both *RAB13* and *NET1* RNAs showed a prominently biased distribution toward the invasive front in leader cells. This bias was not exhibited by the *GAPDH* RNA, which serves as an internal control. A highly significant statistical difference was observed across multiple leader cells analyzed (Fig. 7 *E* and *F*, leader cell panels). By contrast, *RAB13* and *NET1* RNA distributions in follower cells were indistinguishable from that of *GAPDH* RNA, centering around the middle of the cell on average (Fig. 7 *E* and *F*, follower cell panels). These results remarkably show that *RAB13* and *NET1* RNAs accumulate at the invasive front of tumors in vivo. Taken together with the functional importance of this phenomenon demonstrated above, these findings reveal an exciting mechanism that could be exploited to mitigate cancer cell invasion.

## Discussion

We investigated here the localization of the *RAB13* and *NET1* RNAs during 3D collective invasion. We developed a multicellular spheroid model of collective cancer cell invasion and found that both RNAs exhibit a unique localization pattern consisting of a striking accumulation at the front of leader cells in invading strands. This localization requires the microtubule cytoskeleton, laminin association, and integrin engagement. This study identifies and characterizes a previously unappreciated localization of RNAs during collective invasion of cancer cells in a 3D setting. Importantly, RNA accumulation at the invasive front is required for efficient collective invasion and also occurs in in vivo tumors, supporting the generality and functional relevance of the described mechanism.

Multicellular tumor spheroid models have been used to study tumor cell biology in a 3D context and provide insights not observed in simpler 2D monolayers (37). The breast cancer cells we employ here have characteristics of epithelial-to-mesenchymal transition (EMT), such as low E-cadherin expression and up-regulation of EMT transcription factors, features which are typically associated with a loss of cell–cell connections and adoption of single-cell invasion modes (38). Interestingly, however, we find that these low levels of E-cadherin expression are necessary to support the ability of cells to form collective, multicellular invasive strands. Additionally, only a specific window of E-cadherin expression can mediate this effect since high E-cadherin expression impedes invasion, in line with the more traditional role of E-cadherin as a metastasis and invasion suppressor (39). This balance is reminiscent of the notion of “partial EMT” (40, 41) and of the multiple potential roles played by E-cadherin during the metastatic cascade (42). Indeed, cells exhibiting partial EMT phenotypes tend to invade as clusters held together by cell–cell adhesions instead of as single cells (43). The fact that, in the case described here, invasion is additionally induced by serum withdrawal suggests that the migratory pattern adopted by cells is not only influenced by the intrinsic cellular genetic makeup, or the composition of the microenvironment, but is also affected by soluble chemical signals received by cells.

The use of this collective 3D model system has revealed an aspect of cell-specific RNA regulation not appreciated thus far in 2D systems. Specifically, we show that in both in vitro and in vivo settings, RNAs prominently localize at the front of leader cells in invasive strands, while they are only diffusely distributed in follower cells. Leader and follower cells can differ in multiple ways, including their dependence on signaling regulators, their energetic potential, and genetic mutational heterogeneity (44–46). While these factors could contribute to the observed differences in RNA localization phenotypes, we also show that contact with the extracellular matrix, in particular laminin association, is required for RNA accumulation at the front of leader cells. We cannot currently discern whether leader cells produce or reorganize preexisting laminin; however, in other systems leader fibroblasts have been shown to produce and align a fibronectin matrix to promote cancer cell migration (47, 48). Additionally, laminin-5 is frequently found in invading edges of epithelial tumors, where it is positively correlated with invasiveness and poor patient survival (49). With regard to how laminin association could lead to increased RNA accumulation, we think it might be relevant that localization of protrusion-enriched RNAs is promoted by increased actomyosin contractility on stiffer extracellular substrates (6). In this 3D system, the increased laminin concentration observed at the invasive tip could be indicative of denser or stiffer substrate organization. Indeed, stronger forces are exerted by leader cells, and higher traction values have been observed near long, thin cellular protrusions, which may correspond to invasive regions (50–52). We therefore speculate that increased laminin at the invasive tip underlies higher tension and leads to polarized localization of RNAs preferentially within leader cells.

Our findings further demonstrate that RNA accumulation at the invasive front is important to support invasion. We consider that this functional effect is likely mediated by local translation of RNAs at invasive edges. Indeed, *RAB13* RNA is efficiently translated in actively protruding regions of cells on 2D surfaces, and peripheral translation and activation of RAB13 is required for cell migration (11, 12, 24, 26). RAB13 can affect cell migration through multiple mechanisms, including activity-dependent recycling of integrins and modulation of actin-binding proteins at the leading edge (53–55). Similarly, peripheral translation of NET1, a GEF of the RhoA GTPase, could be envisioned to mediate RhoA activation in leader cells and enhance collective invasion (44).

Our finding that the use of antisense oligonucleotides can disrupt the localization of specific RNAs within leader cells and mitigate invasion suggests a potential opportunity for intervention during tumor progression and cancer cell dissemination. Indeed, antisense oligonucleotides are being employed successfully in therapeutic applications (56). However, currently, efficient targeting and sustained effects can be attained when targeting cells of the central nervous system (57, 58). Methodologies to achieve tumor-specific delivery, in combination with chemistries ensuring efficient uptake and sustained action (59–61), will facilitate the exploration of altering subcellular RNA distributions as a therapeutic approach.

## Materials and Methods

### Imaging and Image Analysis.

Images of all fixed fluorescence in situ hybridization (FISH) and immunofluorescence (IF) samples were obtained using a Leica SP8 confocal microscope equipped with a HC PL APO 63× oil CS2 objective. Z-stack images through the entire cell, spheroid, or tissue section volume were obtained, and maximum intensity projections were used for subsequent analysis. Single-cell FISH image analysis was performed using the RDI Calculator (RNA Distribution Index Calculator) (62).

Bright-field time-lapse images of overnight spheroid invasion were obtained on an Olympus IX81 microscope equipped with a 10× (numerical aperture [NA] of 0.3) objective and an Okolab full environmental enclosure with control of temperature at 37 °C, humidity, and atmospheric air.

Time-lapse images of Citrine–CAAX-labeled spheroids were acquired using a Leica SP8 confocal microscope equipped with a HC PL APO 20× dry CS2 objective at constant 37 °C and atmospheric air. Imaging began upon the addition of low-serum media or upon blocking antibody addition. The 488 nm laser line was used for illumination, and Z-stacks through the cell volume were acquired every 15 min over a period of 15.5 h. Maximum intensity projections of the Z-stacks are shown in Fig. 1*A* and Movies S3–S5.

Images of fixed, calcein-stained spheroids were acquired using a Leica SP8 confocal microscope equipped with a HC PL APO 20× dry CS2 objective. Z-stacks through the spheroid volume were obtained, and maximum intensity projections were used for subsequent analysis. Spheroid invasion was quantified by obtaining a complexity value using the TASI software tool (33). Complexity is defined as

$$\text{Complexity}=\frac{{\text{Perimeter}}^{2}}{4\pi \times \text{Area}}.$$

Complexity is the reciprocal value of what is known as the isoperimetric quotient. More invasive spheroids have a higher complexity.

Phase contrast images of spheroids in Matrigel or collagen were acquired using an AMG EVOS fl microscope equipped with a 20× Plan LWD-PH/FL Air objective, 0.40 NA.

### Tongue Tumor Xenograft in Athymic *nu*/*nu* Mice.

All experiments were approved by the National Cancer Institute Animal Care and Use Committee (NIH). Xenograft experiments were conducted based on those in (36). Female athymic (*nu*/*nu*) nude mice (National Cancer Institute at Frederick), 5 wk old and 17 to 25 g, were used in the study. The mice were housed in sterile filter-capped cages and fed and watered ad libitum. Half a million or 1 million HeLa-O3-v cells per animal were submucosally injected in the lateral anterior of the tongue. Animals were fed with a soft dough diet beginning on the day of injection. Tumor growth was monitored by eye on a weekly basis. Two animals, one injected with a million cells and the other with half a million, were randomly selected and euthanized at 2 and 5 wk, respectively. For intravital imaging, mice were anesthetized by an intraperitoneal injection of a mixture of 100 mg/kg ketamine (Vet ONE) and 10 mg/kg xylazine (Anased LA, Vet ONE). The tongues of anesthetized mice were gently pulled out using blunt forceps, and cotton tips were utilized to secure them on the heated stage (37 °C) of a two-photon microscope. Imaging was performed by using an inverted laser-scanning two-photon microscope (MPE-RS, Olympus) equipped with a tunable laser (InSight X3, Spectra Physics). The specimen was excited using a 900 nm laser wavelength, and the emitted light was collected through a 37 °C heated 30× objective (NA of 1.05, UPLSAPO, Olympus) and detected by two GaAs detectors (bandpass filters: blue, 410 to 460 nm, and green, 495 to 540 nm). Ninety to 95 Z-steps (2 µm step size) were acquired using the Olympus Fluoview software and processed with Imaris (Bitplane). For RNA analysis, tissues were collected, immediately embedded in optimal cutting temperature compound (Tissue-Tek, Sakura Finetek), and frozen for FISH analysis.

Additional information on plasmid constructs and lentivirus production, cell culture and generation of stable cell lines, spheroid formation and invasion assay, drug treatments, blocking antibodies, morpholino oligos and delivery, proliferation assay, flow cytometry, immunofluorescence and Western blot, FISH, image analysis, and statistical analysis are described in *SI Appendix*.

## Supplementary Material

Supplementary File

Supplementary File

Supplementary File

Supplementary File

Supplementary File

Supplementary File

## Data Availability

All study data are included in the article and *SI Appendix*.
