# Supplementary material for: Collective cancer cell invasion requires RNA accumulation at the invasive front
Source: Proc Natl Acad Sci U S A. 2020 Oct 15;117(44):27423–34. doi: 10.1073/pnas.2010872117 (PMC7959543; doi:10.1073/pnas.2010872117)
Supplement: Supplementary File [file pnas.2010872117.sapp.pdf]

PNAS
www.pnas.org

Supplementary Information for

Collective cancer cell invasion requires RNA accumulation at the invasive front

George Chrisafis[1], Tianhong Wang[1], Konstadinos Moissoglu[1], Alexander N. Gasparski[1], Yeap Ng[1], Roberto Weigert[1], Stephen J. Lockett[2] and Stavroula Mili[1*]

[1] Laboratory of Cellular and Molecular Biology, Center for Cancer Research, National Cancer Institute, NIH, Bethesda, MD, USA
[2] Optical Microscopy and Analysis Laboratory, Cancer Research Technology Program, Frederick National Laboratory for Cancer Research, Leidos Biomedical Research Inc. for the National Cancer Institute, NIH, Frederick, MD, USA

Stavroula Mili
Email: voula.mili@nih.gov

This PDF file includes:

Materials and Methods
Figures S1 to S9
Legends for Movies S1 to S5

Other supplementary materials for this manuscript include the following:

Movies S1 to S5

www.pnas.org/cgi/doi/10.1073/pnas.20210872117

MATERIALS AND METHODS

Plasmid constructs and lentivirus production

To express Citrine-CAAX, the coding sequence of mCitrine was fused to the C-terminal targeting sequence of K-Ras4B and inserted into the pCDH-CMV lentiviral vector (System Biosciences, cat #CD510B-1).

To express shRNAs targeting luciferase (sh-Control) or E-cadherin (sh-Ecad), the following sequences were cloned into the pLVTHM lentiviral vector (Addgene #12247):
sh-Control: CGTACGCGGAATACTTCGA
sh-Ecad: GGCCTGAAGTGACTCGTAA

To re-express shRNA-resistant E-cadherin in sh-Ecad cells, site directed mutagenesis was used to introduce the following silent mutations (underlined residues; GG CCA GAG GTC ACA AGG AA) within the human E-cadherin coding sequence (corresponding to isoform NM_004360). As a control, instead of E-cadherin, the GFP-nanobody sequence was used. Each coding sequence was cloned into the pENTR1A vector and subsequently transferred into the pINDUCER20 lentiviral vector (Addgene #44012) using the Gateway LR clonase II Enzyme mix (Thermo Fisher Scientific, cat# 11791-020).

Lentivirus was produced in HEK293T cells cultured in DMEM supplemented with 10% FBS and penicillin/streptomycin and grown at 37°C, 5% CO_2. HEK293T cells were transfected with pCDH-CMV, pLVTHM or pINDUCER20 lentivectors, together with packaging plasmids pMD2.G and psPAX2 using PolyJet In Vitro DNA Transfection Reagent (SignaGen) for 48 h. Harvested virus was precipitated with Polyethylene Glycol at 4°C overnight.

Cell culture and generation of stable cell lines

MDA-MB-231 human breast cancer cells (ATCC) were grown in Leibovitz's L15 media supplemented with 10% fetal bovine serum and penicillin/streptomycin at 37°C in atmospheric air. HeLa-O3-v human cancer cells (DNA authenticated; Genetica DNA Laboratories, Inc.) were grown in DMEM supplemented with 10% fetal bovine serum and penicillin/streptomycin at 37°C, 5% CO_2. Cells have been tested for mycoplasma and are free of contamination.

To generate stable cell lines expressing lentiviral constructs, cells were infected with the corresponding lentiviruses at ca. MOI:8. Infected cells were identified through fluorescence expression or selected with Geneticin.

Exogenous expression of E-cadherin was induced by the addition of doxycycline (Thermo Fischer Scientific). The concentration of doxycycline treatment varied as indicated, otherwise 0.1μg/ml was used.

Spheroid formation and invasion assay

To form spheroids, MDA-MB-231 cells were diluted (25,000 cells/ml) and plated as 25μl droplets on the lid of a 15cm culture dish. The lid was inverted onto a dish containing PBS, and aggregate spheroids were allowed to form for 72 h. Spheroids were collected via gentle scraping and spun down. On ice, spheroids were then resuspended in Matrigel (Corning, cat# 354234) and plated in an 8-well chamber slide (30μl/well, 1 plate spheroids/2wells). The bottom of each well was pre-coated with 10μl of Matrigel 30 min prior to plating. Regular media (600μl/well) was added 30 min after plating.

To induce invasion, 2 h after adding media, spheroids were washed once with PBS, and low-serum media (0.1% FBS, 600μl/well) was added. Spheroids were then incubated overnight. To illuminate spheroid borders, calcein dye (Thermo Fischer Scientific, cat# C3100MP, 5μM in PBS) was added, followed by a 20 min incubation at 37°C. Spheroids were fixed with 4% PFA in PBS for 10 min, washed once with PBS and stored in fresh PBS at 4°C until imaging.

Drug treatments

Spheroids were incubated in low-serum media (0.1% FBS) overnight, after which cytochalasin D (10μM) or nocodazole (2.5μM) were added directly to the media for 45 min. A low concentration of nocodazole was chosen in order to not abolish invasion entirely, and enough protrusive strands were retained for analysis. Spheroids were fixed and FISH performed as described.

Blocking antibodies

For blocking antibody experiments, following spheroid invasion, dialyzed anti-laminin-111 blocking antibody (Sigma, cat# L9393, 25μg/ml), anti-ITGB1 blocking antibody (BD, cat# 555002, 25 μg/ml) or anti-rabbit IgG (Sigma, cat# R5506, 25 μg/ml) were added directly to the media. Spheroids were incubated for 3 hrs and fixed for FISH analysis, or incubated overnight, stained with calcein dye and fixed for invasion analysis.

Morpholino oligos and delivery

Antisense morpholino oligonucleotides were synthesized by GeneTools, LLC and delivered into cells either by adding EndoPorter(PEG) (GeneTools, LLC) or through the

use of Vivo-Morpholinos, which can pass directly through cell membranes due to a covalently-linked delivery moiety. A combination of two oligos were used for RAB13 and NET1, at a final concentration of 20 μM. Sequences used are as follows:
Control: 5'-CCTCTTACCTCAGTTACAATTTATA-3'
RAB13: 5'-TCTTTCACTTCCTCAATTCATTCCT-3' and
5'-CCTTCCTTTCCTCCTCCCTCTCTTC-3'
NET1: 5'-TCCCTCTTGCATTTCAGACAACACT-3' and
5'-GACAAAACTACTCTCTTTTCCTCTC-3'

Proliferation assay

Cells were plated in a 24-well plate for approximately 8 h (6,000 cells/well), after which the media was replaced and Vivo-Morpholinos (20μM) were added directly to each well. Cells were counted every 24hrs for 5 days from triplicate wells.

Flow cytometry

For flow cytometry, cells were plated and induced with doxycycline (0.1 μg/ml) for 72 h. To detach cells, they were treated with EDTA (5mM) in PBS for 15 min at 37°C. Cells were then resuspended in flow cytometry buffer (PBS containing Ca^{2+}, Mg^{2+}, and 2% fetal bovine serum) and incubated for 30 min with an antibody diluted in the same buffer. Incubation at 37°C for 1 h following EDTA treatment was included in one trial with no noticeable difference. The antibodies used were anti-E-cadherin (R&D Systems, cat# FAB18381P, 5μl) and anti-mouse IgG (R&D Systems, cat# IC0041P, 5μl) conjugated to phycoerythrin for flow cytometry detection. For analysis, the median fluorescence intensity of E-cadherin was divided by the median intensity of IgG for each sample.

Immunofluorescence and Western blot

For single-cell IF, cells were plated onto collagen IV-coated glass coverslips (10μg/ml) in low-serum media (0.1% FBS) for 1.5 h, then treated with cytochalasin D and nocodazole (same concentration/duration as spheroids). Cells were fixed with 4% PFA in PBS for 15 min, permeabilized with 0.2% Triton X-100 in PBS for 4 min, blocked with 5% fetal bovine serum in PBS for 1 h, and incubated with anti-α-tubulin (Sigma-Aldrich, cat# T6199, 1/500 dilution) for 1.5 h. Secondary antibodies were conjugated with Alexa 647 (Thermo Fisher Scientific), and phalloidin conjugated with Alexa 488 (Thermo Fisher Scientific, cat# A12379, 1/100 dilution) was added to stain F-actin. Nuclei were stained with DAPI, and Fluoromount-G Mounting Media (Thermo Fisher Scientific) was used to mount samples.

For spheroid IF to stain extracellular matrix proteins, upon completion of FISH but prior to adding cell mask stain, spheroids were washed with PBS, blocked with 5% fetal bovine serum in PBS for 1 hr and incubated with anti-laminin (Thermo Fischer Scientific, cat# PA5-22901, 1/100 dilution) conjugated with DyLight 488 or anti-fibronectin (Abcam #ab45688, 1/100 dilution) for 1.5 h. Spheroids were then mounted as described in the FISH protocol. For Ki-67 staining, spheroids were fixed and stained with anti-Ki67 antibody [SP6] (Abcam cat# ab16667, 1/250 dilution).

For Western blotting, cells were plated and induced with doxycycline for 72 h. To make lysate from spheroids, after hanging droplets were incubated for 72 h, spheroids were collected, spun down, resuspended in Matrigel and plated in a 12-well dish (60μl/well, 1 plate spheroids/well). Spheroids were cultured in either normal or low-serum media (0.1% FBS) overnight. To collect spheroids, they were washed once with PBS and a pre-chilled solution of cell harvesting buffer (Trevigen, cat#3448-020-01) in PBS was added to degrade the Matrigel, for 1.5 h on ice at 4°C. For each sample, two wells of spheroids were combined into an Eppendorf tube, spun down (1000xg, 1 min) and resuspended in lysis buffer. The following antibodies were used for detection: anti-E-cadherin mouse clone 36 (BD Transduction Laboratories, cat # 610182, 1/1000 dilution), anti-GAPDH rabbit monoclonal 14C10 (Cell Signaling Technology, cat# 2118, 1/2000 dilution).

Fluorescence in situ hybridization (FISH)

For single-cell FISH, HeLa-O3-v cells were plated in low-serum (0.1% FBS) media on fibronectin-coated glass coverslips (5μg/ml) for 4-5 h and then fixed with 4% PFA for 20 min. The View RNA ISH Cell Assay kit (Thermo Fischer Scientific) was used to perform FISH, according to the manufacturer's instructions. The following probe sets were used: human RAB13 #VA1-12225, human NET1 #VA1-20646 and human RHOA #VA6-14829. CellMask stain (Thermo Fischer Scientific) was used to identify cell outlines, and DAPI was used to stain nuclei. ProLong gold antifade reagent (Thermo Fischer Scientific) was used to mount samples. Image analysis and quantification of RNA distributions was performed using the RDI calculator (62).

For FISH of spheroids, following overnight invasion, spheroids were washed once with PBS. To fix spheroids and dissolve the Matrigel, a pre-chilled solution of 4% PFA/cell harvesting buffer in PBS was then added for 2 h, on ice at 4°C. If the matrix was not fully dissolved after 2 h, additional harvesting buffer in PBS was added overnight. FISH was performed as described above, but with 5 min wash steps throughout.

For FISH of tissue sections, OCT-embedded frozen tissues were cut (20μm) and put onto silanated glass slides, dried at -20°C for 2 hr and stored at -80°C in an airtight container. The RNAscope Multiplex Fluorescent V2 Assay (Advanced Cell Diagnostics) was used to perform FISH, according to the manufacturer's instruction. For the pretreatment of cryosections, the provided "Fresh-frozen sample preparation and pretreatment" protocol was followed, with one modification: frozen cryosections were immediately fixed with 4% PFA in PBS at RT for 1 h. The following probe sets were used: hsRAB13 (cat# 810241), hsNET1 (cat# 810461), hsGAPDH (cat# 442201-C2), mmGAPDH (cat #442871-C3) and negative control probes (cat# 320871). Opal 520, 570 and 690 fluorophores (Akoya Biosciences, cat#FP1487001KT, FP1488001KT, FP1497001KT, 1:1500 dilution for all) were used for probe detection.

Imaging and image analysis

For spheroids, z-stacks of magnified protrusive cell strands were obtained in addition to images of the spheroid body. RNA distributions were then analyzed using a two-step process. First, a custom MATLAB script identified individual RNA spots based on the ratio of local signal intensity peaks to the surrounding background. The script then determined the distance of each spot to two user-defined "edges" in the image. For leader cells, they were the front of the nucleus ("nuclear edge") and protrusive tip of the cytoplasm ("invasive edge"). We consider as "invasive edge" the tip of the most extended protrusion even though secondary, side protrusions might exist. Any RNA spots located within nuclei were omitted. RNA distances were then further analyzed using a custom RStudio script. For each cell, the length between edges (L) was calculated:

$$L = \frac{1}{n}\sum_{i=1}^{n} Nd_i + Id_i$$

where n equals the total number of RNAs in a cell. The mean normalized distance (M) of each RNA type, RAB13 (or NET1) and RHOA, to the invasive edge was then determined:

$$M_{RNA_1} = \frac{1}{n}\sum_{i=1}^{n}\frac{Id_i}{L} \qquad M_{RNA_2} = \frac{1}{n}\sum_{i=1}^{n}\frac{Id_i}{L}$$

where n equals the total number of each respective RNA type in a cell. M is a number between 0 and 1, with values closer to 0 representing an RNA distribution that is biased toward the invasive edge. Across multiple cells, a probability density function (using the kernel density estimation) that described the mean normalized distance of each RNA type was plotted, and a Wilcoxon matched-pairs sign rank test statistically compared the distributions. The same analysis workflow was used to analyze RNA distributions in

follower cells, characterized as the cell immediately trailing a leader cell. In this case, the edges were defined as the front of the follower cell nucleus and back of the leader cell nucleus.

Analysis of RNA distributions in tissue sections was performed in a similar manner. The imaging of tissue sections focused on xenograft edges, characterized by an interface between the xenograft and normal mouse tissue. Along these edges, strands of cells with distinct leader and follower cells were observed protruding into the surrounding tissue. We presumed these strands to have an invasive phenotype based on prior data (36). Protrusions were randomly selected and imaged, without viewing the RNA channel prior to imaging. Leader and follower cells were manually identified, with leader cells being the frontmost cell in a protrusion and follower cells being a cell within the same strand that had a well-defined cytoplasm by human GAPDH signal. RNA distributions in leader and follower cells were quantified using the same process as spheroids. Within a cell, the distribution of a localized RNA was compared to GAPDH, which is randomly distributed throughout the cytoplasm.

ECM protein distributions (laminin and fibronectin) were quantified from IF images in two steps. First, leader cell outlines were manually traced in ImageJ. Outlines began at the front edge of the nucleus on one side of the cell, followed the membrane curvature to the protrusive tip, and ended at the front edge of the nucleus on the other side. Signal intensity values of either laminin or fibronectin at all points along the outline were exported and analyzed using a custom RStudio script. Briefly, intensity values within a protrusion were scaled to have a maximum value of 4095, the highest pixel value in the dynamic range of a 12-bit image. Scaled data across protrusions was aligned along the x-axis based on the x-coordinate of each protrusive tip. Intensity values were binned by first separating the data into segments containing roughly 10-15 individual values, and then computing the average of these values. The size and numerical limits of each bin were kept consistent across all protrusions, and the bin containing the protrusive tip was assigned an x-axis value of 0. Due to protrusions being different lengths, a mean intensity and 95% confidence interval were calculated and plotted for bins which contained data from greater than 50% of the protrusion sample size.

Statistical analysis

The normality of distributions was assessed using the D'Agostino & Pearson test or Shapiro-Wilk test (GraphPad Prism). Normally distributed datasets were analyzed using parametric statistical tests. Datasets deviating from a normal distribution were analyzed

using nonparametric tests. The statistical test used in each case, as well as the sample sizes and number of replicates are indicated in the respective figure legends.

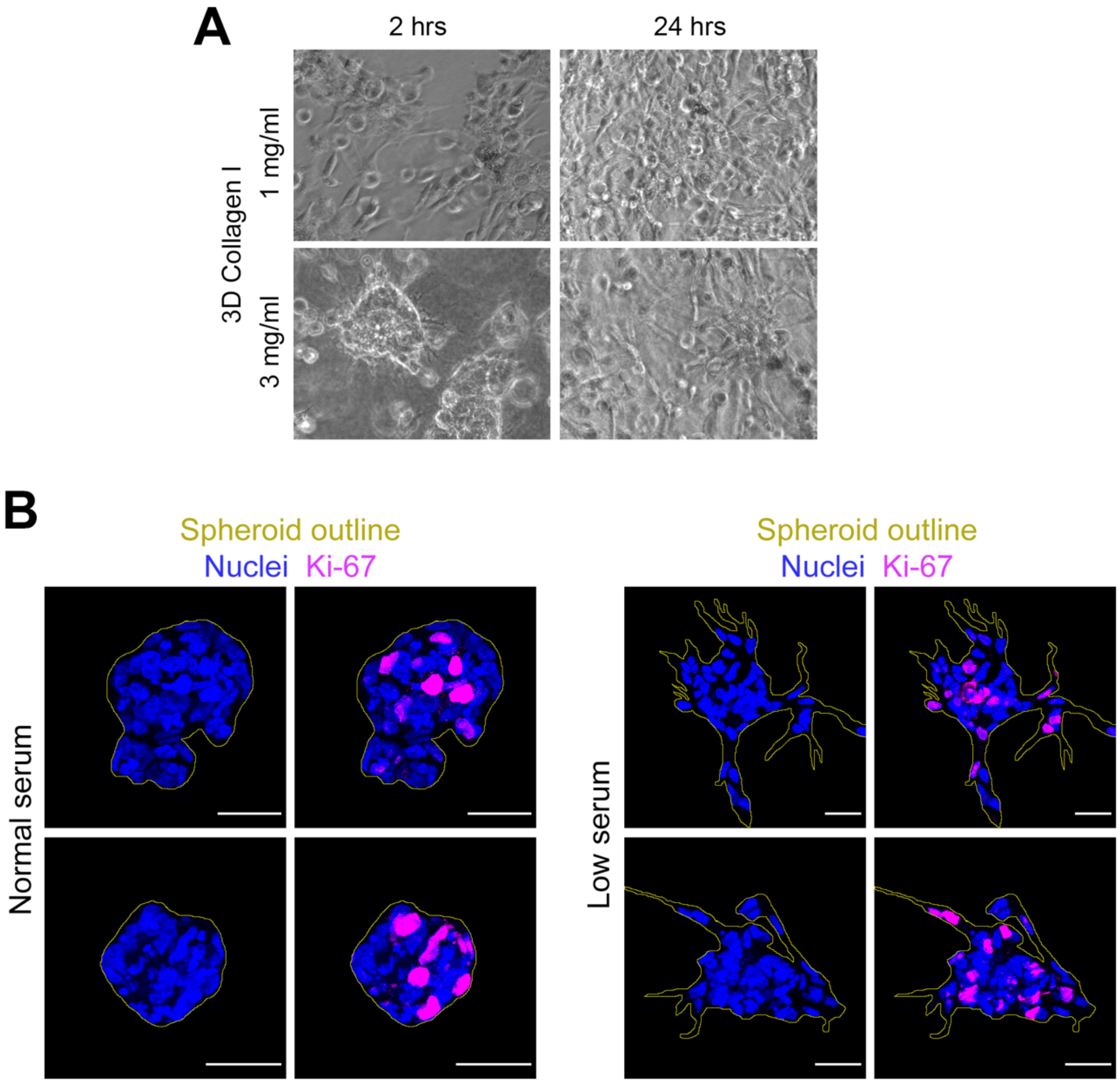

Fig. S1. Spheroid invasion in Collagen I or Matrigel. A. MDA-MB-231 spheroids were embedded into 3D collagen I matrix, of the indicated concentrations, and observed by brightfield illumination 2 or 24 hrs after. **B.** MDA-MB-231 spheroids were embedded in Matrigel, grown in normal serum (10% FBS) and switched, or not, to low serum (0.1% FBS) for ca. 10 hrs. Spheroids were stained to detect the proliferation marker Ki-67. Serum reduction induces invasion but does not halt cell proliferation. Scale bars: 30 μm.

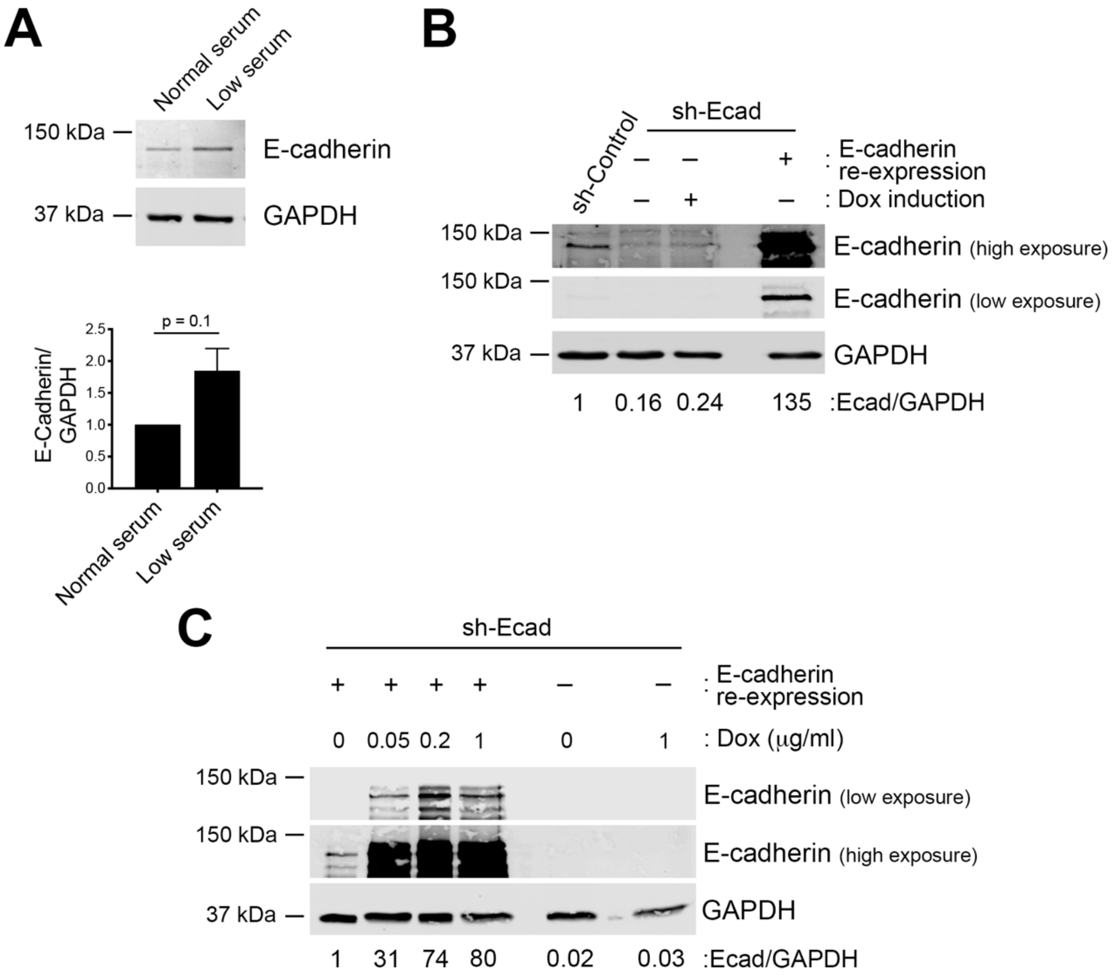

Fig. S2: E-cadherin expression levels. A. Western blot analysis of E-cadherin expression from spheroids in Matrigel grown under normal or low serum. n=3. p-value by Mann Whitney test. **B, C.** Western blot analysis of E-cadherin expression from the indicated cell lines. Note that leaky expression of exogenous E-cadherin (i.e. in the absence of doxycycline; panel B, lane 4; panel C, lane 1) leads to much higher levels compared to the endogenous. Most of the expressed protein does not traffic to the surface (compare with Figure 1B). Different exposures and sample combinations are shown to capture the wide spread of expression levels.

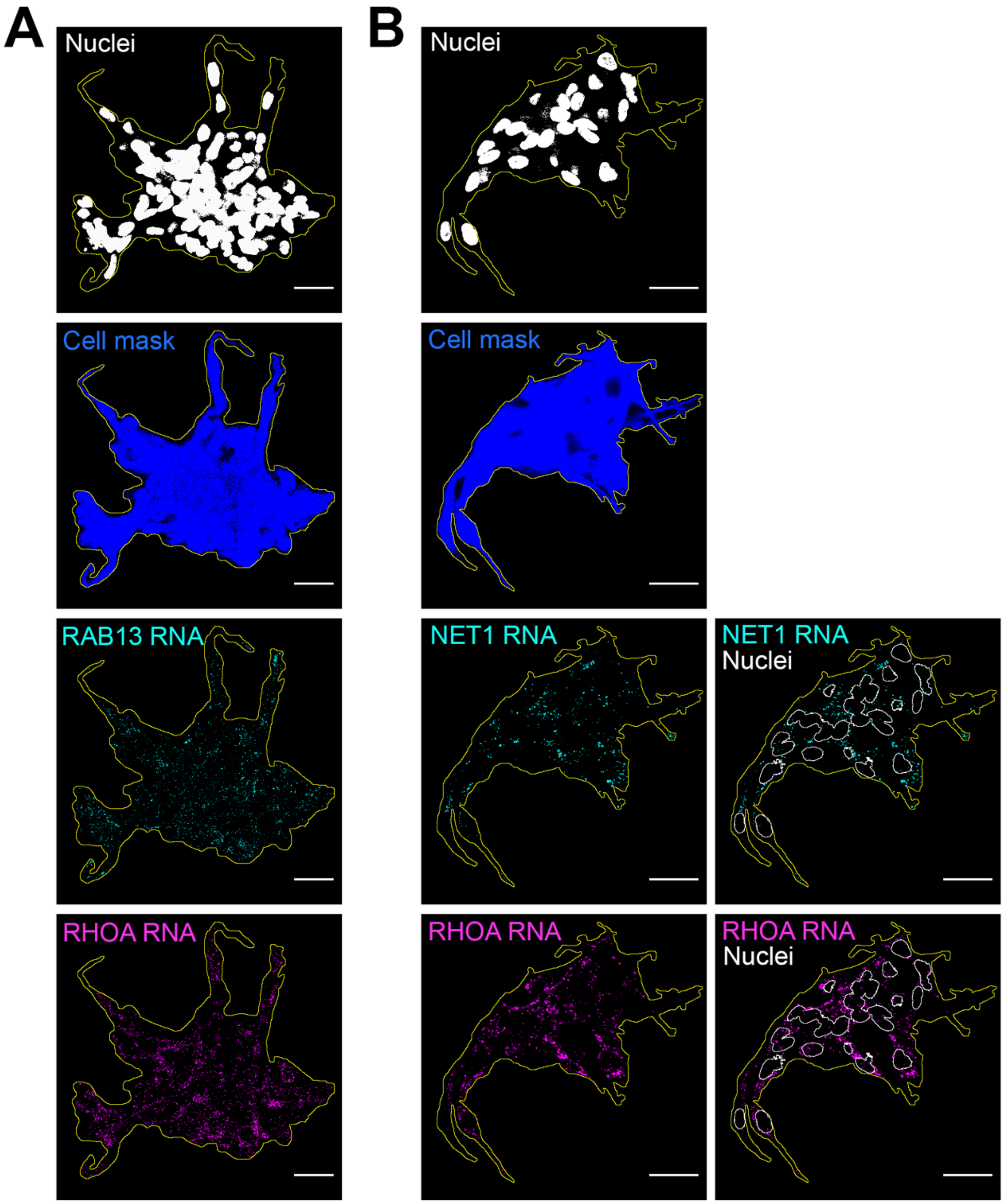

Fig. S3: RNAs can be detected throughout 3D spheroid structures. In situ hybridization of invasive MDA-MB-231 spheroids in Matrigel, detecting the *RAB13* and *RHOA* RNAs (**A**) or the *NET1* and *RHOA* RNAs (**B**). Cell mask staining is used to delineate the spheroid outline. A single optical slice through the middle of the spheroid is shown. Note that RNAs can be imaged throughout the spheroid body. **B.** (Right panels) Outlines of nuclei are overlaid on RNA channels. Note that the majority of RNA signal is detected within cytoplasmic areas. Scale bar: 30 μm.

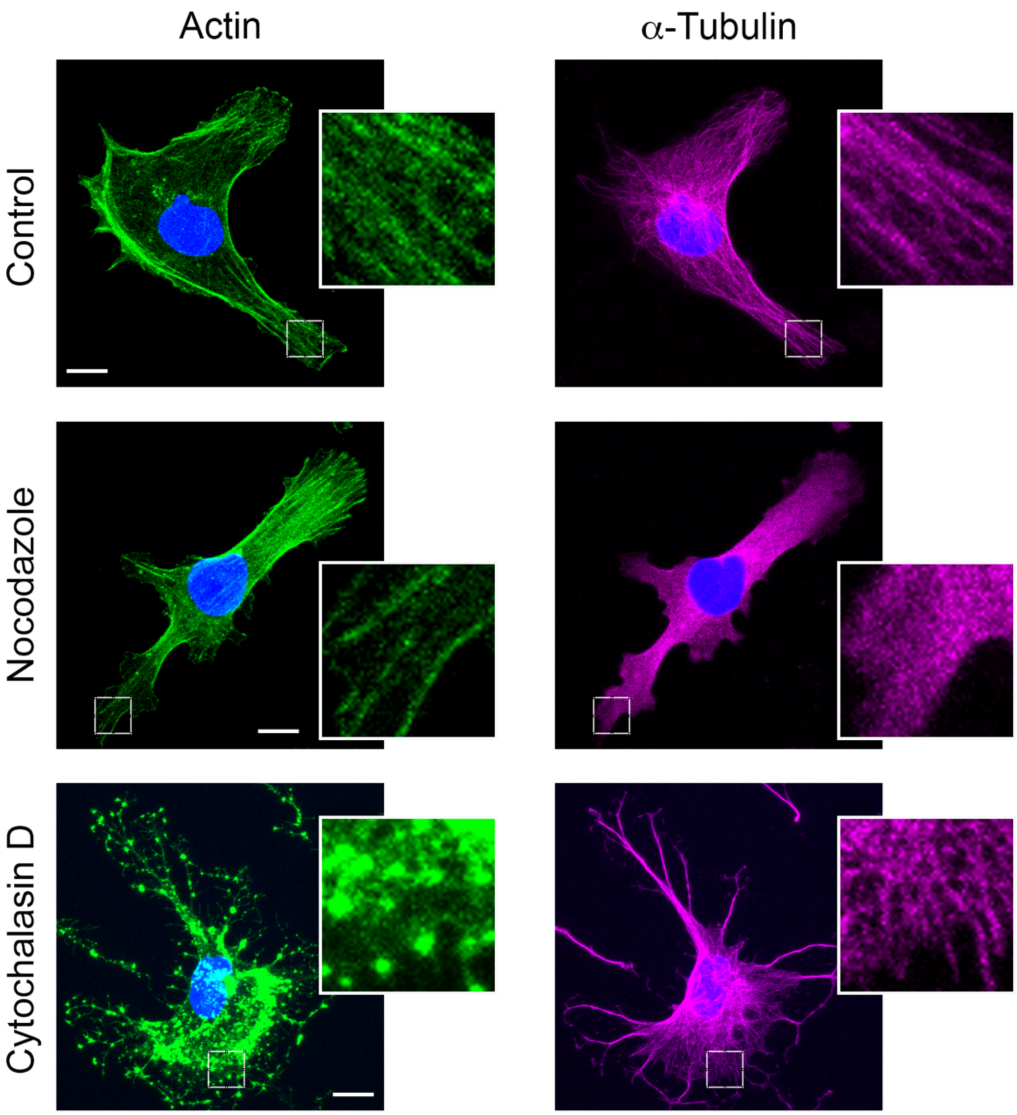

Fig. S4: Disruption of actin and microtubule cytoskeleton. Fluorescence imaging of the actin and microtubule cytoskeleton to assess the effect of the indicated compounds. F-actin visualized through phalloidin staining, microtubules through anti-tubulin immunofluorescence. Boxed regions are enlarged in the insets. Scale bars: 10 μm.

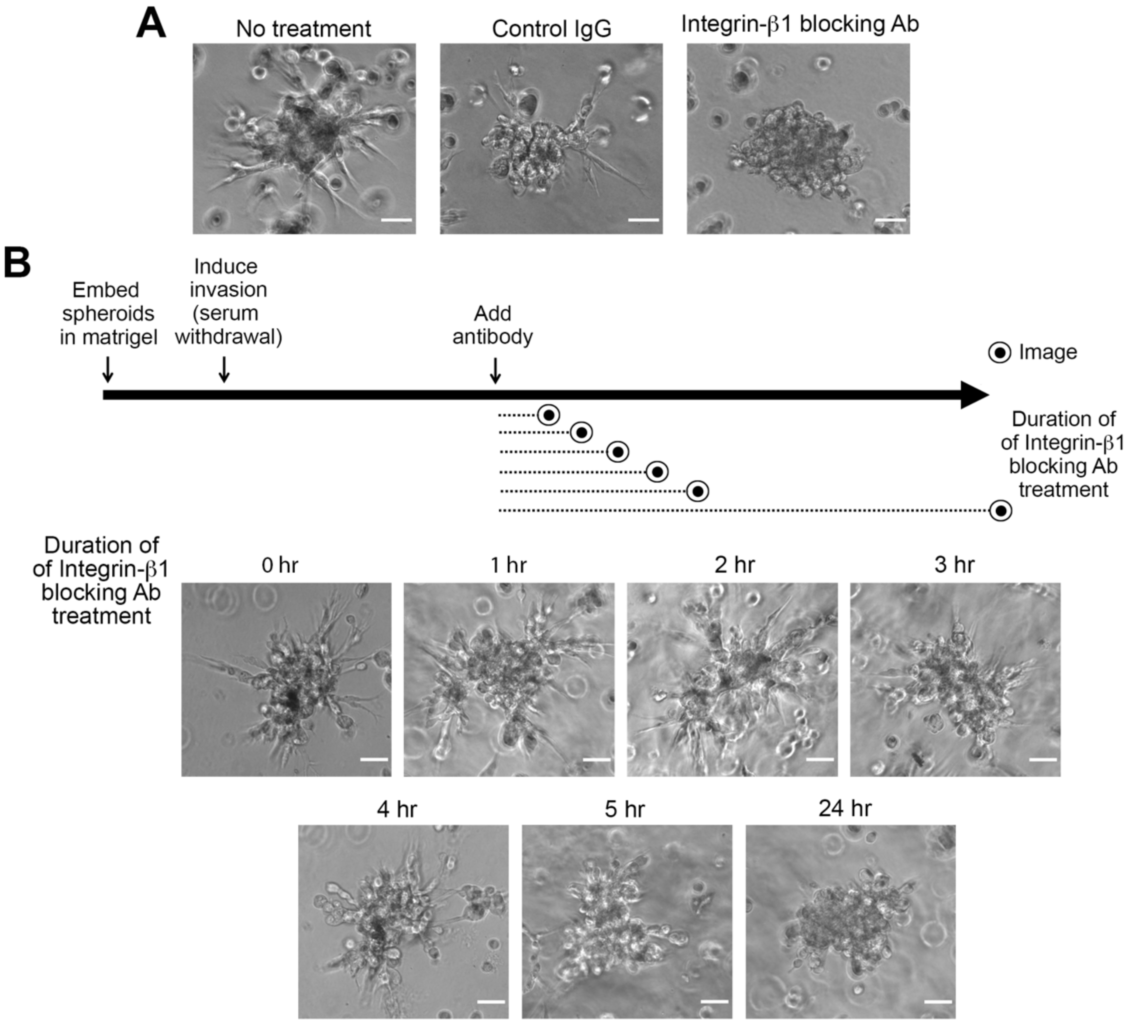

Fig. S5: Prolonged integrin blocking severely blocks invasion. A. Representative brightfield images of invasive spheroids treated overnight with the indicated antibodies. Note that Integrin-blocking Ab prevents invasion. **B.** Invasive spheroids were treated with Integrin-blocking Ab for increasing amounts of time. At 3 hr, inhibition begins to be detectable but leader cells can still be identified (see also videos S4 and S5). Scale bars: 100 μm.

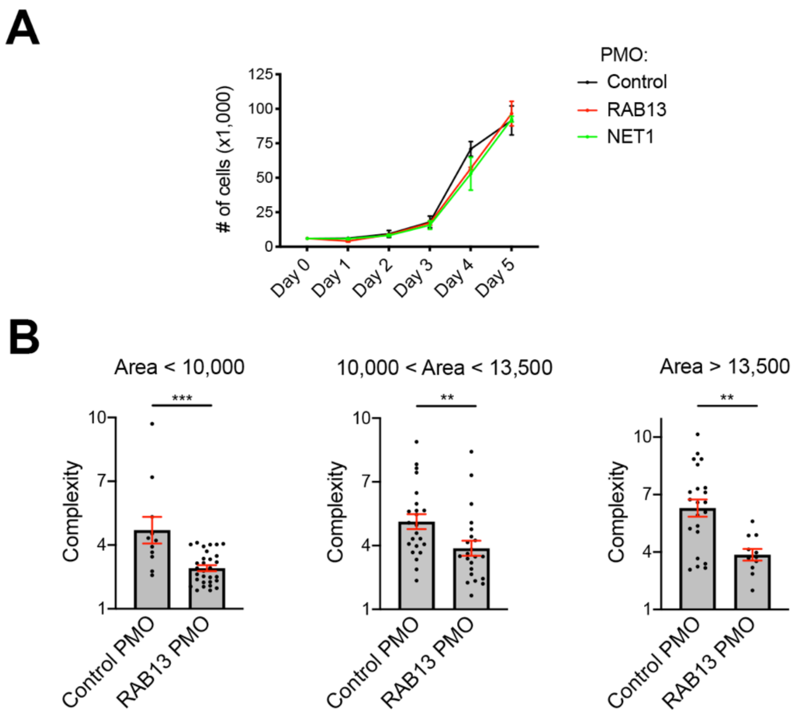

Fig. S6: *RAB13* RNA mislocalization does not affect cell growth and disrupts invasion regardless of spheroid size. A. Growth curves of cell treated with the indicated PMOs. **B.** Complexity values of invasive spheroids from cells treated with control or RAB13 PMOs (from Figure 6C). Spheroids were divided into groups of increasing size based on area measurements. p-values: **<0.01, ***<0.001, by Mann-Whitney test. Error bars: standard error.

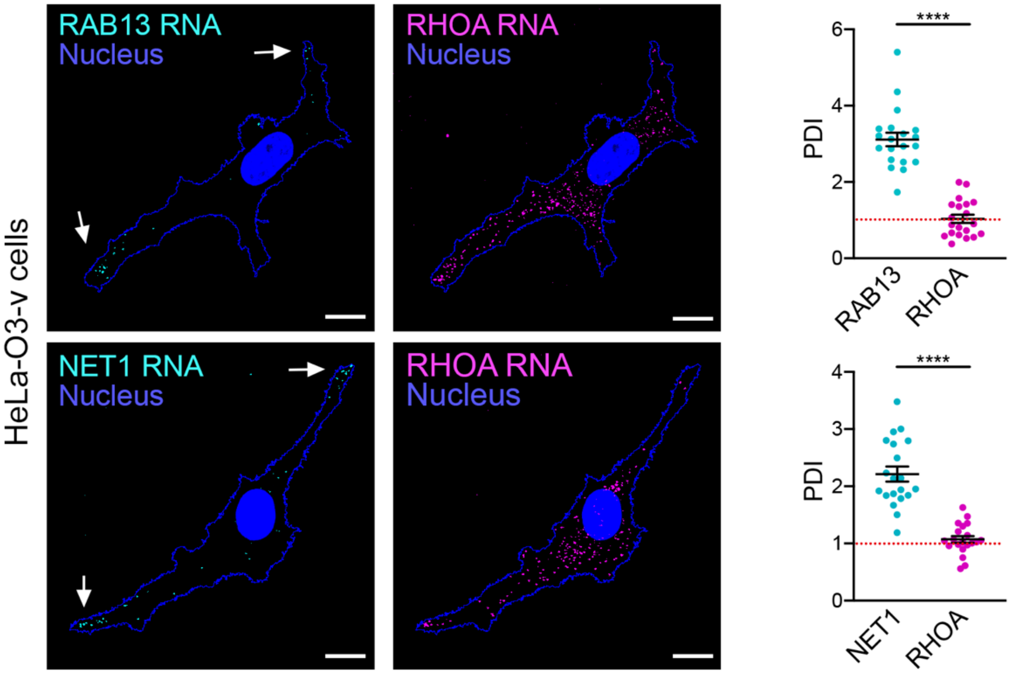

Fig. S7: *RAB13* and *NET1* RNAs are localized at protrusions of HeLa-O3-v cells. In situ hybridization to detect the indicated RNAs in HeLa-O3-v cells. Arrows point to *RAB13* and *NET1* RNA accumulation at peripheral protrusions. *RHOA* RNA is used as internal control, diffuse RNA. Corresponding graphs depict PDI values measuring peripheral RNA distribution. PDI value of 1 (dashed red line) indicates a diffuse distribution. n=20. p-values: ****<0.0001 by Student's t-test. Error bars: standard error. Scale bars: 10 μm.

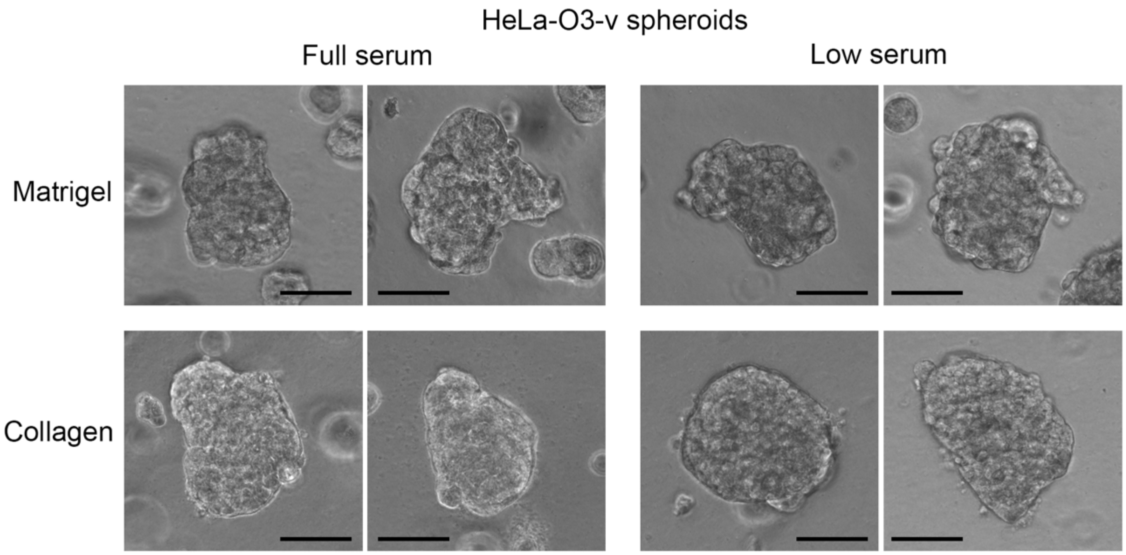

Fig. S8: HeLa-O3-v cells do not form invasive spheroids in Matrigel or Collagen I. Brightfield images of HeLa-O3-v spheroids embedded in Matrigel or Collagen I (3mg/ml). Similar to the conditions used for MDA-MB-231 spheroids, they were grown either in full serum (10%) media or switched to low serum (0.1%) overnight. Spheroids do not exhibit an invasive phenotype under any condition. Scale bars: 100 μm.

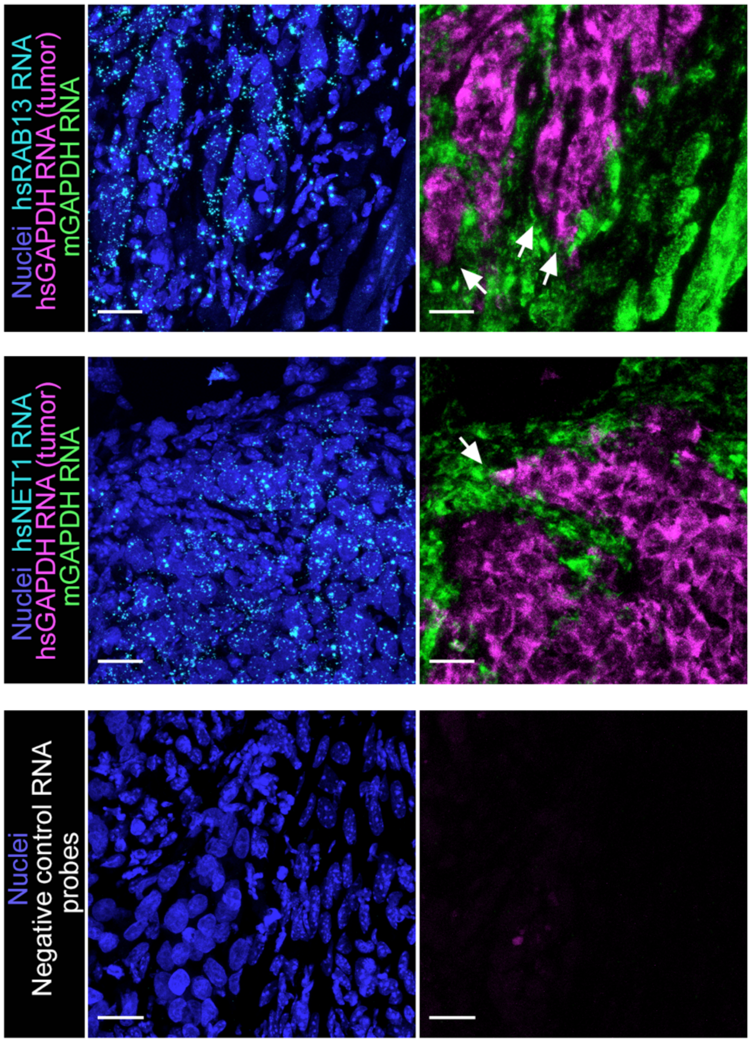

Fig. S9: Specificity of FISH staining of tissue sections. FISH staining of tongue tissue sections (20μm thick) with probes against the indicated RNAs or negative control probes. The panels detecting specific human or mouse RNAs are the same as those in Figure 7C and 7D and are presented again here for comparison to the negative control. Scale bars: 20 μm

Movie S1 (separate file): Brightfield imaging of non-invasive spheroid. Time lapse imaging of multicellular MDA-MB-231 spheroid embedded in Matrigel and maintained in media with regular serum. Imaging started ca. 5 hrs after embedding. Time stamp in Hours:Minutes.

Movie S2 (separate file): Brightfield imaging of invasive spheroid. Time lapse imaging of multicellular MDA-MB-231 spheroid embedded in Matrigel. Spheroid was maintained in media with regular serum for ca. 5hrs and then switched to low serum media. Imaging was initiated ca. 30min after media change. Time stamp in Hours:Minutes.

Movie S3 (separate file): Fluorescence imaging of invasive spheroid. Time lapse imaging of multicellular MDA-MB-231 spheroid expressing Citrine-CAAX. Spheroid was embedded in Matrigel in media with regular serum for ca. 5hrs and then switched to low serum media. Imaging was initiated ca. 30min after media change. Time stamp in Hours:Minutes.

Movie S4 (separate file): Fluorescence imaging of invasive spheroid in the presence of integrin-blocking antibody. Time lapse imaging of multicellular MDA-MB-231 spheroids expressing Citrine-CAAX. Spheroids was embedded in Matrigel in media with regular serum for ca. 4 hrs, switched to low serum media for ca. 8 hrs and integrin blocking antibody was added. Imaging was initiated ca. 45min after antibody addition. Time stamp in Hours:Minutes. Scale bars: 50 μm.

Movie S5 (separate file): Fluorescence imaging of invasive spheroid in the presence of control antibody. Time lapse imaging of multicellular MDA-MB-231 spheroids expressing Citrine-CAAX. Spheroids was embedded in Matrigel in media with regular serum for ca. 4 hrs, switched to low serum media for ca. 8 hrs and control antibody was added. Imaging was initiated ca. 45min after antibody addition. Time stamp in Hours:Minutes. Scale bars: 50 μm.
